# Rhodopsins: An Excitingly Versatile Protein Species for Research, Development and Creative Engineering

**DOI:** 10.3389/fchem.2022.879609

**Published:** 2022-06-22

**Authors:** Willem J. de Grip, Srividya Ganapathy

**Affiliations:** ^1^ Leiden Institute of Chemistry, Department of Biophysical Organic Chemistry, Leiden University, Leiden, Netherlands; ^2^ Radboud Institute for Molecular Life Sciences, Radboud University Medical Center, Nijmegen, Netherlands; ^3^ Department of Imaging Physics, Delft University of Technology, Netherlands

**Keywords:** membrane protein, photoreceptor, retinal protein, visual pigments, optogenetics, ion pumps, microbial, eukaryotic

## Abstract

The first member and eponym of the rhodopsin family was identified in the 1930s as the visual pigment of the rod photoreceptor cell in the animal retina. It was found to be a membrane protein, owing its photosensitivity to the presence of a covalently bound chromophoric group. This group, derived from vitamin A, was appropriately dubbed retinal. In the 1970s a microbial counterpart of this species was discovered in an archaeon, being a membrane protein also harbouring retinal as a chromophore, and named bacteriorhodopsin. Since their discovery a photogenic panorama unfolded, where up to date new members and subspecies with a variety of light-driven functionality have been added to this family. The animal branch, meanwhile categorized as type-2 rhodopsins, turned out to form a large subclass in the superfamily of G protein-coupled receptors and are essential to multiple elements of light-dependent animal sensory physiology. The microbial branch, the type-1 rhodopsins, largely function as light-driven ion pumps or channels, but also contain sensory-active and enzyme-sustaining subspecies. In this review we will follow the development of this exciting membrane protein panorama in a representative number of highlights and will present a prospect of their extraordinary future potential.

## Introduction

**FIGURE 1:**
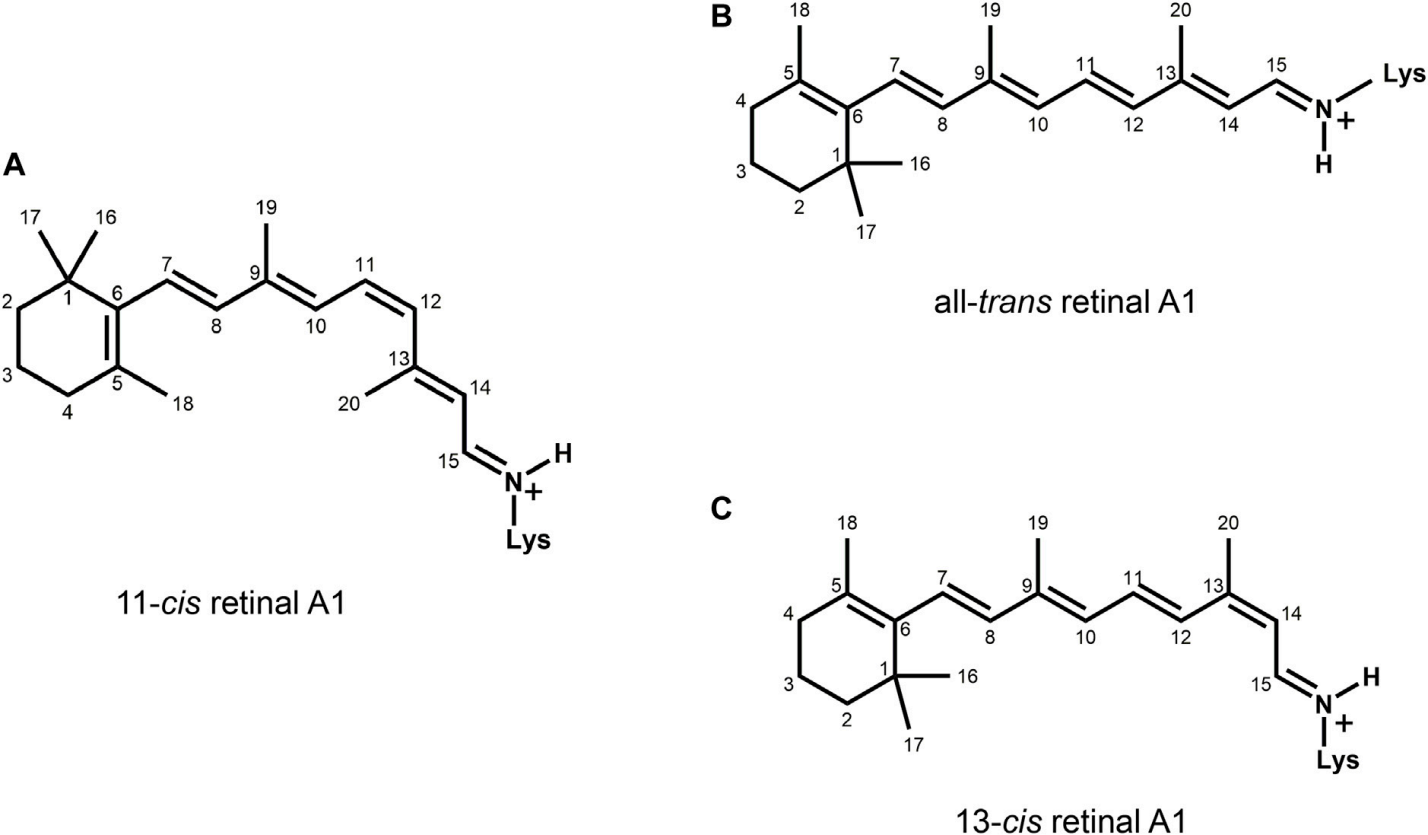
Chemical structures of the most common chromophore configurations in the rhodopsin families. The type-2 pigments contain an 11-*cis*, 15-*anti* retinylidene Schiff base of retinal A1 **(A)** in the “dark state” (or “ground state” in photophysical terminology), which is photo-excited into the all-*trans* configuration. The Type-1 pigments contain the all-*trans* configuration **(B)** in the “dark state.” This is photo-excited into the 13-*cis*, 15-*anti* configuration **(C)**, which thermally relaxes and re-isomerizes, returning to the ground state. The ring-polyene chain orientation is different for type-2 (6-s-*cis*) and type-1 (6-s-*trans*) rhodopsins.

The first member and eponym of the rhodopsin family was identified in the 1930s as the visual pigment of the rod photoreceptor cell in the animal retina (Tansley, 1931; Wald, 1935). It turned out to be a membrane protein, owing its photosensitivity to the presence of a covalently bound chromophoric group. This photosensitive group, derived from vitamin A, was appropriately coined retinene, later officially renamed as retinal (Wald, 1935; Morton and Goodwin, 1944; Morton and Pitt, 1957). The visual pigments harboured a special conformer of this polyene compound, in casu the 11-*cis* configuration (Hubbard and Wald, 1952; Hubbard et al., 1971) (Figure 1). Upon photo-activation the chromophore was converted into the all-*trans* configuration, which triggered a sequel of conformational changes in the protein, leading to its active state (Wald, 1953; Morton and Pitt, 1957; Dartnall, 1962a). Eventually the chromophore was released as all-*trans* retinal (Dartnall, 1962c; Wald, 1968; Hubbard et al., 1971; Bridges, 1972). Surprisingly, in the 1970s a microbial counterpart of this protein was discovered in the archaeon *Halobacterium salinarum* (at the time referred to as *Halobacterium halobium*), which also harboured retinal as a chromophore, and was named bacteriorhodopsin (Oesterhelt and Stoeckenius, 1971). This membrane protein, however, contained the all-*trans* configuration, which upon photo-activation was converted into the 13-*cis* configuration (Smith et al., 1985; Oesterhelt, 1998). The resulting active state of the protein in this case thermally decayed in a sequel of steps whereby the chromophore eventually was thermally re-isomerized into the all-*trans* configuration returning to the original starting state (Oesterhelt, 1998; Lanyi, 2004).

Since their discovery a photogenic panorama unfolded, where up to date new members and subspecies with a variety of light-driven functionality have been added to these families. The animal branch, categorized as type-2 rhodopsins, turned out to form part of the major subclass in the superfamily of G protein-coupled receptors (Bennett et al., 1982; Kühn, 1984; Crescitelli, 1991; Hargrave and McDowell, 1992). Currently they have diversified into at least eleven groups (Opn1–Opn9, R-group, Cn-group) most of which are essential to multiple elements of light-dependent animal sensory physiology. Depending on the animal species, they can be located in multiple tissues next to the eye (Terakita, 2005; Davies et al., 2015). Meanwhile, the microbial branch was named as type-1 rhodopsins, which largely function as light-driven ion pumps or channels, but also contain sensory-active and enzyme-sustaining subspecies (Oesterhelt, 1998; Spudich et al., 2000; Ernst et al., 2014; Leung and Montell, 2017; Nagata and Inoue, 2022). The most recent addition to the microbial rhodopsins is the heliorhodopsin family, which is remarkably different from the type-1 family in their inverted orientation in the membrane, with the N-terminal now residing in the intracellular compartment (Pushkarev et al., 2018; Shihoya et al., 2019; Kovalev et al., 2020b; Rozenberg et al., 2021; Chazan et al., 2022). The physiological function of this new family has not become very clear as of yet.

In this review we follow the historical development of this exciting membrane protein panorama in a representative number of highlights and present a prospect of their extraordinary future potential. We broadly outline their functional diversity and physiological relevance, as a comprehensive description is outside the scope of this review. A large number of excellent reviews on the rhodopsin families have been published, many of which we have referred to where appropriate, along with the most relevant early and recent papers. We refrain from presenting many molecular details, and therefore we refer to the following more recent reviews (DeGrip and Rothschild, 2000; Hofmann, 2000; Spudich et al., 2000; Hofmann et al., 2009; Yizhar et al., 2011; Palczewski and Orban, 2013; Ernst et al., 2014; Imamoto and Shichida, 2014; Inoue et al., 2014; Deisseroth, 2015; Hofmann and Palczewski, 2015; Brown and Ernst, 2017; Bando et al., 2019; El Khatib and Atamian, 2019; Dowling, 2020; Kandori, 2020; Kwon et al., 2020; Baillie et al., 2021; Moraes et al., 2021; Rozenberg et al., 2021; Bondar, 2022; Broser, 2022; Brown, 2022; Khelashvili and Menon, 2022; Nagata and Inoue, 2022).

This review presents a historical perspective and is therefore organized according to the landmark discoveries or progress in the field. In the following sections, we first discuss milestone studies and the common elements of the type-2 and type-1 rhodopsins, followed by individual subsections presenting typical elements for the type-2 and type-1 family, respectively. For the interested reader, we have compiled additional relevant citations in tables accompanying every section.

## Discovery

The discovery and identification of rhodopsins was governed by their spectral properties. Since they all absorb photons in the visible spectrum, careful visual observations were the cornerstone for these early studies.

### Type-2 Family

**FIGURE 2:**
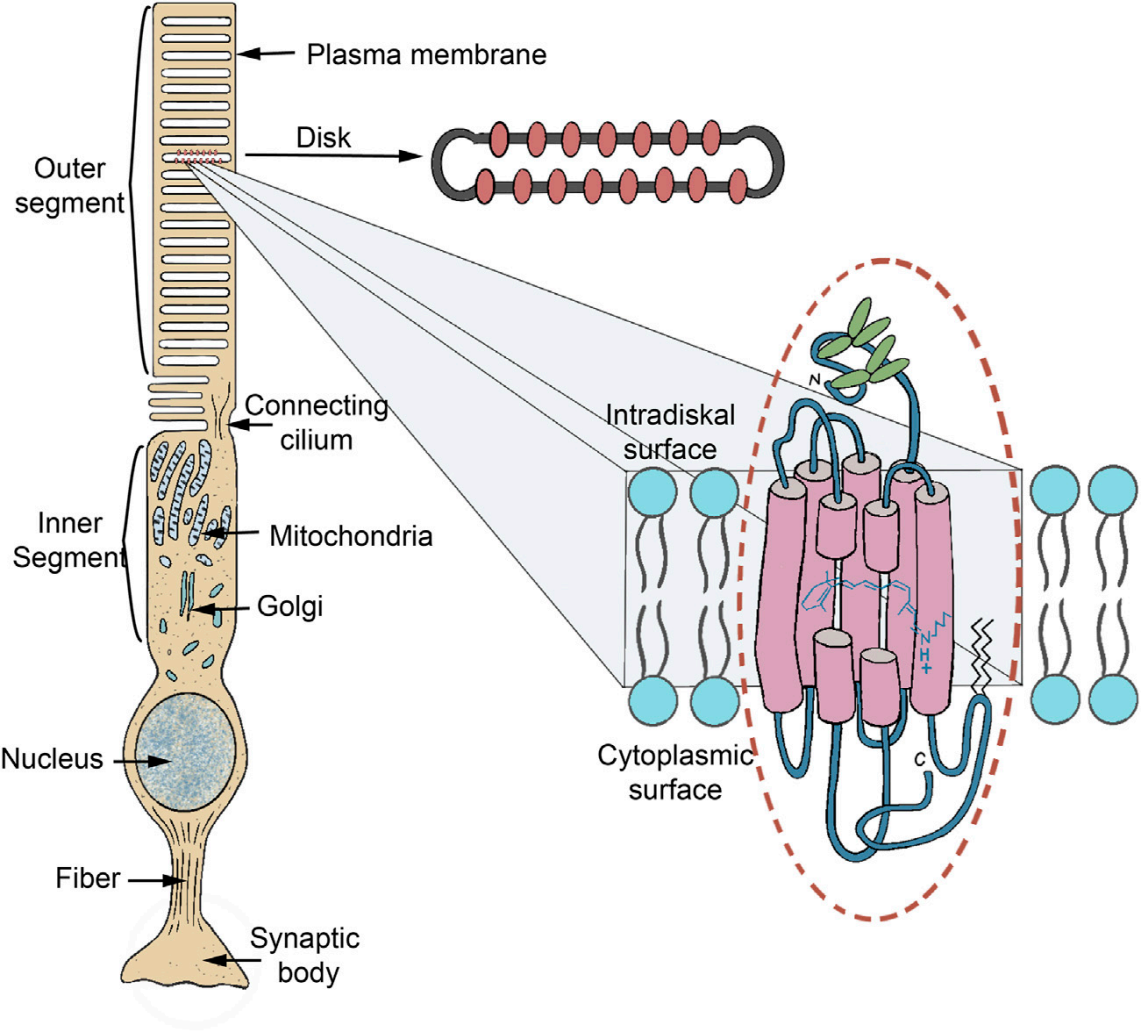
Schematic of a vertebrate rod photoreceptor cell (scotopic vision), zooming in on the location of the rod visual pigment rhodopsin. The rod outer segment (ROS), a ciliary outgrowth, is densely filled with isolated flattened vesicles (discs) which contain rhodopsin as the major (ca 90% w/w) membrane protein. The vertebrate visual pigments are therefore also designated as “ciliary rhodopsins.” Other disc membrane proteins are involved in signal propagation, stabilization of the disc shape and communication with the plasma membrane (PM). The phospholipids in the disc membrane have an exceptionally high content (ca 40%) of highly unsaturated fatty acids (22:6∞3) (Daemen, 1973). The discs are continuously generated at the base of the ROS as invaginations of the PM, then are nipped off and move upwards. After 7–10 days they reach the top of the ROS, which is pinched off in a circadian rhythm and degraded in the adjacent retinal pigment epithelium (RPE) (Young, 1976). The vertebrate cone photoreceptor (photopic vision) is organized in a similar fashion, except that the “discs” remain continuous with the PM as invaginations and are not pinched off. The organization of invertebrate visual photoreceptors is roughly similar, but the photoreceptive membranes are organized as numerous microvilli in rhabdomeric structures (Warrant and McIintyre, 1993) and their rhodopsins are also designated as rhabdomeric visual pigments. Only the classical visual pigments (Opn1, Opn2 and R-gene families) are organized in these specialized cellular outgrowths. All other type-2 and all type-1 pigments are targeted to the PM or an eyespot and form only a small part (up to several percent) of that membrane protein population.

Rhodopsin, the founding father of the type-2 family was first identified as the visual pigment of the rod photoreceptor cell. In the 19th century, groundbreaking research on vision by Müller, Boll and Kühne led to the visual perception, that light capture occurred in the distal part of the human retina (Figure 2), in particular the outer segments of the photoreceptor cells (Müller, 1855; Boll, 1877; Ewald and Kühne, 1878). The typical red color of this tissue disappeared upon illumination, which was termed “bleaching,” and could to some extent be regenerated upon subsequent dark adaptation of the isolated eyecup. As of the 1930s it became apparent that a membrane-bound protein in the rod photoreceptor cell was responsible for the red color (Tansley, 1931; Bliss, 1948; Wald, 1953). This protein was named rhodopsin, after the ancient Greek words ροδεοσ (rhodeos, rose-coloured) and οψισ (opsis, which appropriately can be translated as sight or eyes). It was found to owe its spectral properties to a covalently bound cofactor, eventually named retinal (Wald, 1935, 1953, 1968; Hubbard et al., 1971). Subsequently, it was discovered that the cone photoreceptors in the vertebrate retina harboured closely related visual pigments (Morton and Pitt, 1957; Dartnall, 1962c; Mustafi et al., 2009). Thereafter, it became known that the invertebrate retina applied structurally very similar, but photochemically slightly differently operating visual pigments (Hara et al., 1967; Suzuki et al., 1993; Gärtner, 2000). Similar “bi-stable” pigments in fact are also active in the vertebrate retina, like the well-known melanopsins (Provencio et al., 1998; Kumbalasiri and Provencio, 2005). Another highlight was the growing insight that the visual pigments form part of the superfamily of G protein-coupled receptors (Kühn, 1984; Hargrave and McDowell, 1992; Palczewski and Orban, 2013). As a matter of fact, rhodopsin is the cornerstone of the major subfamily in this widespread receptor family.

### Type-1 Family

In the early 1970s, fascinated by the dark-purple colonies of the salt lake thriving archaeon *Halobacterium salinarum*, Oesterhelt reported the surprising discovery that an intrinsic membrane protein was dominating purple patches in the cellular membrane of this archaeon and also harboured retinal as the chromophoric cofactor (Oesterhelt and Stoeckenius, 1971; Oesterhelt and Hess, 1973). At that time archaea were considered a subfamily of bacteria, and Oesterhelt coined the name bacteriorhodopsin (BR). Surprisingly, it was discovered that bacteriorhodopsin functions as a light-driven outward-directed proton pump, creating a proton-motive force enabling the cellular ATP-synthase complex to supply the cell with metabolic energy in the form of ATP (Oesterhelt et al., 1991). While bacteriorhodopsin is the dominant photoreceptor in *Halobacterium salinarum*, this archaeon eventually turned out to harbour several related photosensitive proteins, both with ion transport and sensory functions (Oesterhelt, 1998). Since the 1990s this field exploded, with more strains, including eukaryotic organisms like algae and fungi, and other functionalities being revealed every year (Béjà et al., 2000; Spudich et al., 2000; Brown, 2004; Rozenberg et al., 2021; Broser, 2022; Nagata and Inoue, 2022). More recently even viral rhodopsins have been discovered (Philosof and Béjà, 2013; Bratanov et al., 2019; Zabelskii et al., 2020). The overall structure and photochemistry of these pigments are very similar, and they are now considered to be a primary factor in marine phototrophy and solar energy conversion (Kirchman and Hanson, 2013; Gómez-Consarnau et al., 2019).

## Spectral and Structural Properties, and Solubilization

**FIGURE 3:**
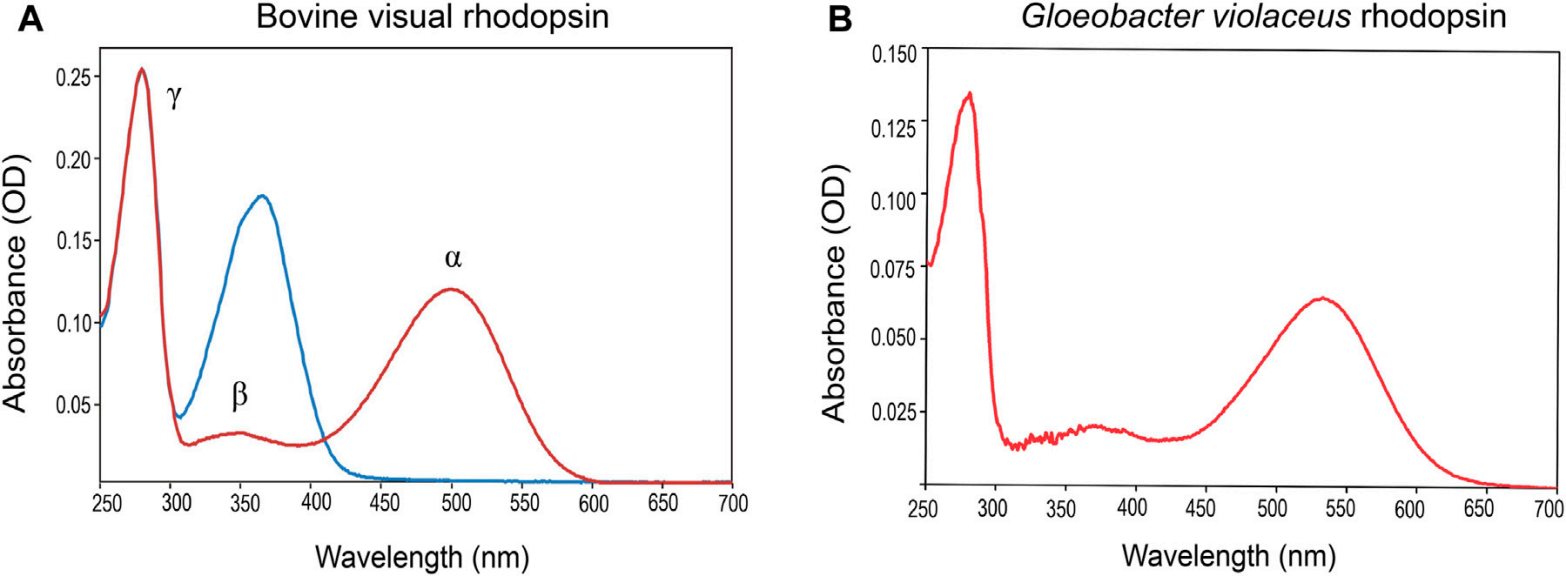
Typical dark state absorbance spectra (red curves) of a purified type-2 **(A)** and type-1 **(B)** pigment. Both spectra exhibit a major peak (α-band) and a small satellite (β-band), both originating in the chromophore, and a γ-band near 280 nm, mainly originating in protein residues. The α-band derives from the whole conjugated polyene system (S0-S1) (cf. Figure 1), while the β-band derives from a smaller segment, and its intensity also depends on the torsion in the polyene chain. Upon short illumination of the monostable type-2 pigment **(A)** in the presence of hydroxylamine, the liberated retinal is converted into retinaloxime (blue curve). The Meta state of bistable type-2 pigments, also reacts with hydroxylamine generating retinaloxime, but usually quite slowly. Short illumination of type-1 pigments **(B)** in the presence of hydroxylamine hardly affects the photocycle and the return to the ground state. However, upon prolonged illumination hydroxylamine will slowly attack photo-intermediates, mainly M and N, releasing retinaloxime.

**FIGURE 4:**
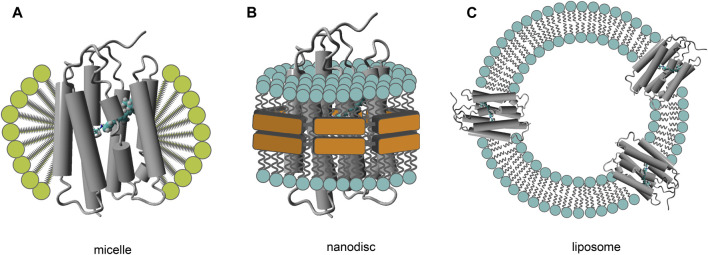
Membrane mimics for purified membrane proteins. Schematics of the micellar **(A)**, nanodisc **(B)** and vesicular (**C**; proteoliposome) organization are displayed. Note that the relative dimensions are not to scale: diameters vary from 10–50 nm for the micelles and nanodiscs, and from 100 nm up to 10 µm for the liposomes. Several amphipatic components functioning as bilayer-stabilizing agents in the nanodiscs have been generated (MSP derivatives from lipoproteins, synthetic amphipols and SMAs, respectively), and are still under further development. Purification of the protein in a detergent environment generates the classical micellar state **(A)**. Because the thermal stability of membrane proteins in the micelles is generally reduced, often (phospho)lipids are added (bicelles). Alternatively, membrane proteins can be transferred into the bilayer membrane of a nanodisc **(B)** or liposome **(C)**. Membrane proteins can be directly (amphipol or SMA nanodiscs) or under very brief detergent exposure (MSP nanodiscs) transferred from the native membrane into nanodiscs, and the classical purification techniques can be applied upon the resulting nanodisc population. Liposomes offer a broader selection for the lipid population, and are used in vectorial transport studies and in AFM, FTIR and solid-state NMR spectroscopy. However, they are less suitable in optical spectroscopy because of their larger dimension, resulting in strong light scattering.

The spectral properties of all rhodopsins were discovered by visual observation, thanks to their absorbance of photons in the visible spectrum (350–750 nm). Accurate recording of their absorbance spectra was complicated in the spectrophotometers available at that time, due to the intense scattering of light by the rhodopsin containing membrane fragments isolated from host cells. Strong chemical reagents or alkaline conditions could dissolve these fragments, but with concomitant denaturation of the proteins and loss of their native spectral properties (bleaching). In the 1950s synthetic surface-active agents, termed detergents, became available, that were able to solubilize these membrane proteins in smaller mixed detergent-lipid-protein micelles, which strongly reduced light scattering (Hallett et al., 1991). Strong detergents like SDS still led to denaturation and release of retinal, but milder detergents were developed to avoid rapid partial unfolding at lab temperature or below. Accurate recording of absorbance spectra could then be established in detergent solutions. If some scattering still remained, or other visible light material interfered, difference spectroscopy was established by recording spectra before and after illumination in the presence of hydroxylamine and taking a difference spectrum. Hydroxylamine captures the released retinal as retinaloxime, which absorbs outside the main absorbance band of most rhodopsins (Wald and Brown, 1953; Hubbard et al., 1971; Kropf, 1975). This usually provides an accurate profile of the main absorbance band or at least the absorbance maximum. (Figure 3). A more recent and elegant approach is to insert a membrane protein into small nanodiscs (Civjan et al., 2003; Borch and Hamann, 2009; Ritchie et al., 2009) (Figure 4). This also strongly reduces light scattering and has the important advantage of embedding the protein in the more stabilizing lipid bilayer environment (Banerjee et al., 2008; Tsukamoto et al., 2011; Zhou and Cross, 2013; Ganapathy et al., 2020). Nanodiscs can be generated using either lipoproteins and membrane scaffold protein derivatives (MSPs) or small synthetic polymers of the amphipol or styrene-maleic acid copolymer family (SMAs) (Knowles et al., 2009; Popot et al., 2011; Hoi et al., 2021). For MSPs usually a brief detergent solubilization step is still required, while SMAs can extract the protein directly from the membrane, but have a smaller pH-profile (Shirzad-Wasei et al., 2015; Dörr et al., 2016; Kopf et al., 2020; Ueta et al., 2020).

The spectral profile of rhodopsins in the visible and near-UV region is very similar (Figure 3). It consists of the most red-shifted main absorbance band or α-band, a smaller β-band, both originating in the bound retinal, and the γ-band near 280 nm, that largely originates in the aromatic residues of the protein part termed “opsin.” In all rhodopsins retinal is covalently linked to a lysine residue in the seventh transmembrane segment (TM7) via a Schiff base (Figure 1) which is mostly protonated. The α-band is strongly red-shifted from the absorbance band of free retinal (maximum around 380 nm). This unusual polar grouping in the middle of a membrane protein is stabilized by the negatively charged “counterion complex,” containing one, two or occasionally three protein residues (mostly Glu/Asp, sometimes Lys or in anion pumps a Cl^**−**^ ion) in a H-bonded network with nearby residues and bound water molecules (Lanyi, 2004; Ernst et al., 2014; Gerwert et al., 2014; Nomura et al., 2018). Thus, the excitation energy in this retinylidene moiety is strongly reduced, compared to free retinal, which results in a red-shift of the absorbance profile. The magnitude of the red-shift strongly depends on the structure of the H-bonded network and counterion complex involving variable electrostatic interactions with the protonated Schiff base and to a lesser extent on the properties of protein residues in the opsin binding pocket (Lesca et al., 2018; Nikolaev et al., 2020; Shen et al., 2021; Shtyrov et al., 2021; Church et al., 2022a). By modifying these elements Nature created the spectacular broad variance in the spectral profile of rhodopsins, allowing them to cover the entire visible region.

The three-dimensional (3-D) structure of rhodopsins has been extensively investigated by classical electron diffraction on 2-D crystals and X-ray crystallography on large 3-D crystals, by solid-state NMR spectroscopy on membrane fragments and more recently by X-ray free electron lasers (XFEL) on small crystals (Schertler and Hargrave, 1995; Palczewski, 2012; Ladizhansky, 2017; Smith, 2021). Cryo-electron microscopy (Cryo-EM) has been traditionally performed on micellar solutions, but has also evolved to include nanodiscs (Maeda et al., 1991; Bertazolli-Filho et al., 2001; Hasegawa et al., 2018; Zhao et al., 2019; Zhang M. et al., 2021). Only solid-state NMR can be directly applied to membrane suspensions, but overall, there is quite good agreement between the various approaches. The overall structure is quite similar for all rhodopsin families, with the main scaffold consisting of seven closely packed transmembrane α-helices, which creates a tightly fitting binding pocket lined by a lysine residue to covalently bind retinal (Figure 2). The protein N- and C-terminal stretch reside at the extracellular and intracellular side of the membrane, respectively, except for the heliorhodopsin family where this sidedness is reverted (Pushkarev et al., 2018). However, the packing of the α-helices, the size of the loops connecting the α-helices and of the N-terminal and C-terminal stretches outside the membrane differ significantly between the type-1 and type-2 families.

### Type-2 Family

Most type-2 rhodopsins, and in particular cone visual pigments and invertebrate pigments are very sensitive to at least partial denaturation upon solubilization in detergent solution (Bliss, 1948; Kropf, 1982; Okano et al., 1989). While commercial detergents like Triton X-100, CTAB, LDAO and Emulphogene BC-720 could dissolve the vertebrate rod pigment rhodopsin into mixed micelles with none or only very slow loss of spectral properties at room temperature, for most other pigments only the very mild agent digitonin could be applied (Tansley, 1931; Knudsen and Hubbell, 1978; Okano et al., 1989; Hofmann and Palczewski, 2015). This natural compound, a steroidal glycone extracted from *Digitalis purpurea*, however has the disadvantage that its commercial preparations were quite expensive and did vary in composition and aqueous solubility (Bridges, 1977). Major progress was attained in the 1970s upon development of the alkylsaccharide detergents 1-O-n-β-D-octylglucoside (octylglucoside, OG), nonylglucoside (NG) and dodecylmaltoside (DDM) (Stubbs et al., 1976; DeGrip and Bovee-Geurts, 1979). DDM in particular turned out to maintain thermal stability and spectral and photochemical properties of rhodopsin almost as well as digitonin (DeGrip, 1982; VanAken et al., 1986). Additionally, DDM is well accessible and affordable through organic synthesis, and has therefore become the most popular detergent in the membrane protein field. Also, in case a protein purified in DDM needs to be reconstituted in a lipid bilayer for certain applications (nanodisc or proteoliposome, Figure 4), DDM can be easily extracted via cyclodextrin inclusion (DeGrip et al., 1998). More recently, a large number of novel detergents based upon the structural principle of DDM have been developed, some of which provide better thermal stability or better crystallization conditions for selected membrane proteins than DDM, but all requiring more complex synthesis (Hussain et al., 2016; Nguyen et al., 2018; Ehsan et al., 2020; Urner et al., 2020).

The absorbance band profiles of type-2 rhodopsins are quite similar (Figure 3), but the position of the α-band varies strongly for the visual pigments. The vertebrate rod photoreceptor pigment rhodopsin has quite a broad range in its absorbance maximum (Rh1 subset, 440–520 nm), with fresh-water animals slightly red-shifted and marine animals blue-shifted depending on the depth of their habitat (Locket, 1977; Luk et al., 2016; Musilova et al., 2019). Vertebrate cone pigments cover the entire visible spectrum, and can be divided into four subsets, the long-wavelength (LWS, absorbance maximum range 520–640 nm), green (Rh2, 460–530 nm), blue (SWS2, 400–470 nm), and UV (SWS1, 350–450 nm) sensitive pigments (Crescitelli, 1991; Yokoyama and Yokoyama, 2000; Imamoto and Shichida, 2014). This classification is not only based upon spectral sensitivity, but also upon sequence similarity (Nathans, 1987; Hunt and Collin, 2014; Jacobs, 2018; El Khatib and Atamian, 2019). Invertebrate visual pigments are more scattered over the visible region and can range from 340 nm up to 600 nm (Gärtner, 2000; Katz and Minke, 2009; Tsukamoto and Terakita, 2010). Non-visual animal rhodopsins are scattered over the 340–550 nm region (Leung and Montell, 2017; Pérez J. H. et al., 2019; Moraes et al., 2021).

**FIGURE 5:**
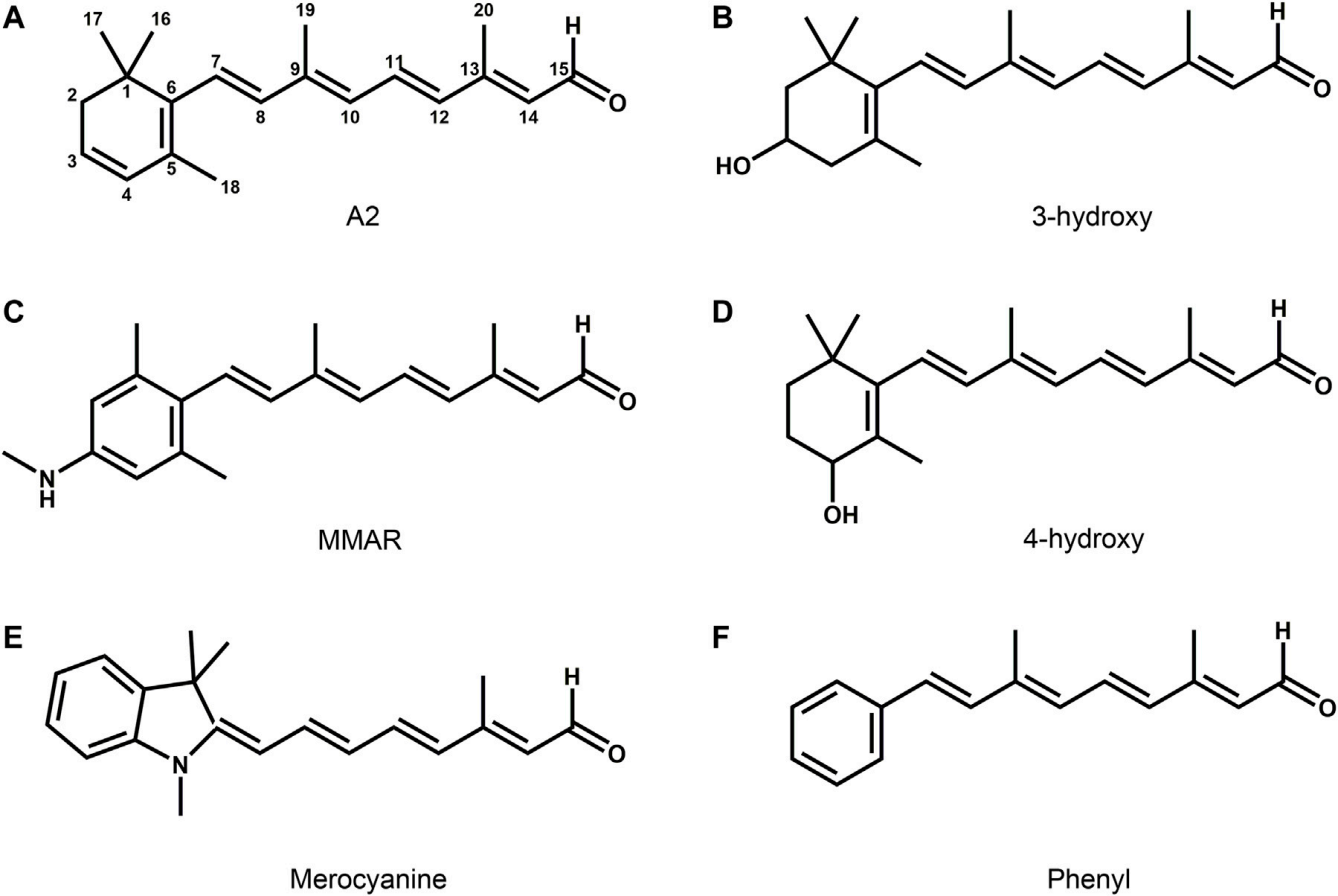
Some uncommon retinal analogs occurring as natural chromophores or in engineered pigment analogs. 3, 4-didehydroretinal (retinal A2, **(A)**) red-shifts the rhodopsin spectrum relative to A1, and is mostly found in fish and amphibian visual pigments. 3-hydroxy- **(B)** and 4-hydroxy- **(D)** retinal A1 induce a blue-shift relative to A1 and are found in the visual pigments of insects and deep-sea shrimps, respectively. Phenylretinal **(F)**, MMAR **(C)** and the merocyanine derivative **(E)** are synthetic analogs, that, respectively, induce a blue-shift **(F)** and the largest red-shifts, observed so far (**(C,E)**; see text). All these analogs bind to the lysine residue in the native opsin binding pocket with a protonated Schiff base.

The spectral properties of the type-2 rhodopsins depend on the 11*-cis* configuration of the retinylidene chromophore. Next to the standard retinal (retinal A1, Figure 1), several natural modifications occur (analogs). In fresh-water and coastal vertebrates 11-*cis* 3-dehydroretinal (retinal A2) has been observed (Figure 5) (Bridges, 1972; Yoshizawa, 1984; Imai et al., 1999). The longer conjugated chain red-shifts the absorbance maximum by 20–40 nm in rod pigments and up to 70 nm in cone pigments, as compared to retinal A1, to compensate for the lower blue light intensity in their habitat (Dartnall, 1962c; Hubbard et al., 1971). These “A2-rhodopsins” are also referred to as porphyropsins. In insects and some other invertebrates, 11*-cis* 3-hydroxy- and 4-hydroxyretinals have been detected (Figure 5) (Vogt and Kirschfeld, 1984; Matsui et al., 1988; Seki and Vogt, 1998). These modifications blue-shift the absorbance maximum by 20–40 nm, as compared to retinal A1 (Sekharan et al., 2011).

While such natural modifications are exploited to modulate the spectral position of a rhodopsin, the most effective approaches to shift the absorbance spectrum of the rhodopsin chromophore away from that of free retinal (380 nm) are protonation of the Schiff base and mutation of selected opsin residues lining the retinal binding pocket. For instance, only the Schiff base in UV absorbing rhodopsins, which absorb in the 350–380 nm region, is not protonated, while in all other classes it is protonated (Kusnetzow et al., 2004; Imamoto and Shichida, 2014). The large variation in the spectral properties in the latter classes is mainly due to the combined inductive effect of opsin binding pocket residues, in combination with H-bonding networks involving water molecules. On top of that, some vertebrate LWS visual pigments have developed a unique mutation (Glu197->His) creating a chloride binding site that effectuates a further 20–30 nm red-shift (Wang et al., 1993).

**FIGURE 6:**
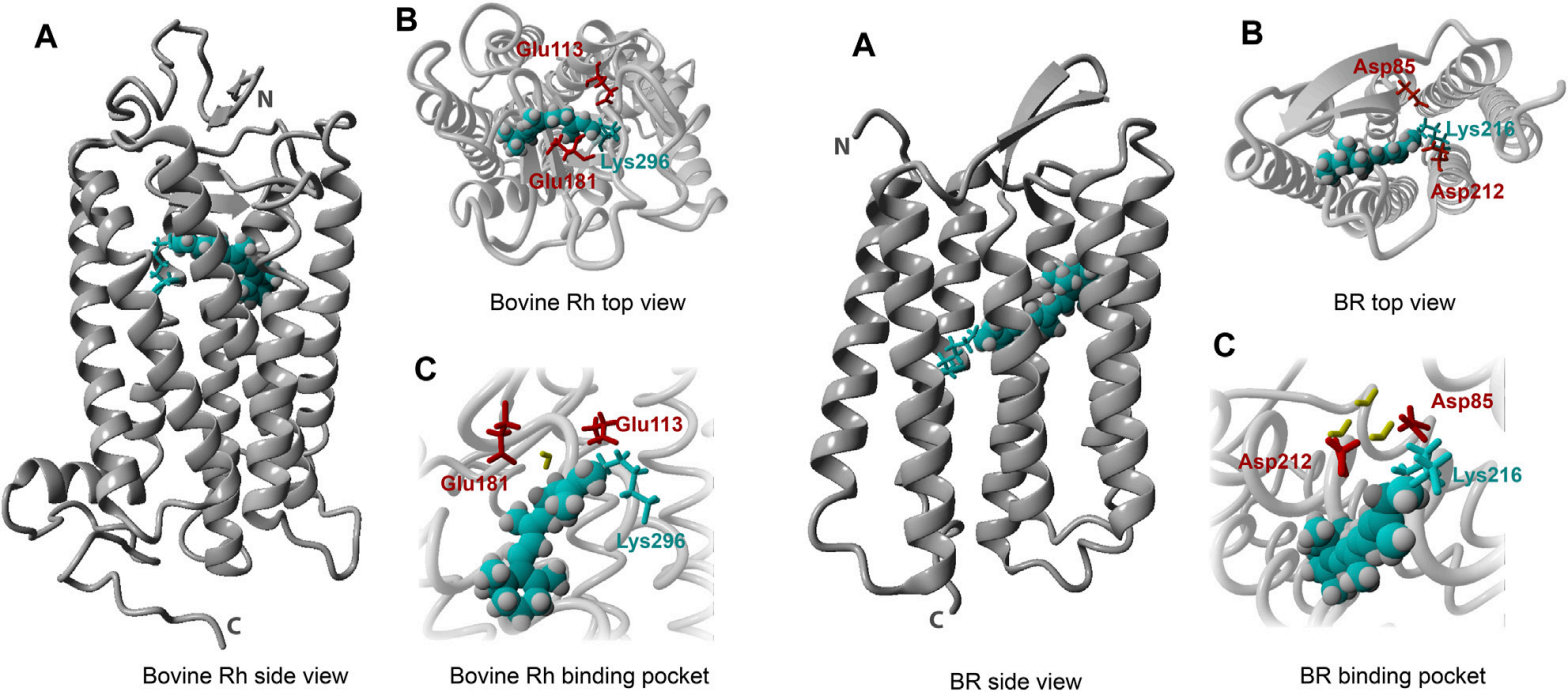
Comparison of structural features of the type-2 and type-1 pigment archetypes bovine rod rhodopsin (left section) and bacteriorhodopsin (right section), respectively. Full crystal structures are presented in **(A)** and **(A)** (pdb 1U19 and 5ZIN), a top view is shown in **(B)** and **(B)** and a binding pocket exposure in **(C)** and **(C)**, respectively. The retinylidene chromophore (cyan) is represented as space-filling spheres, and the retinal binding lysine residue (cyan) is presented as sticks. The two protein residues displayed (red) contribute to the counterion complex stabilizing the pronated Schiff base. The two crystal structures share the seven α-helical transmembrane segment bundle, but the packing of the helices, the location and assembly of the binding pocket and the structure of the chromophore are clearly different.

With respect to structural biology, bovine rod rhodopsin was a forerunner among all animal intrinsic membrane proteins, presenting the first detailed 3-D structure via X-ray crystallography in 2000, with many more to follow (Palczewski et al., 2000; Li et al., 2004; Okada et al., 2004). The seven transmembrane α-helical scaffold surrounding an accessible cofactor binding pocket proved to be the general motif for the entire G protein-coupled receptor family (Figure 6) (Sanchez-Reyes et al., 2017). This feat has stimulated advances in many other research fields, including drug design in the pharmaceutical sciences, study of protein structure-function correlations, and membrane protein-lipid interactions, both from experimental, theoretical and *in-silico* standpoints. Several natural factors concurred to enable this important step forward. First of all, rod rhodopsin is one of the few intrinsic membrane proteins that is available in relatively large quantities in domesticated animals, the most used being cattle (up to 1 mg of rhodopsin per eye), bullfrogs (up to 100 μg per eye) and chick (up to 100 μg LWS cone pigment per eye) (DeGrip et al., 1980; Toba and Hanawa, 1985; Yoshizawa and Kuwata, 1991). After enucleation and proper dark adaptation of the eyes, intact rod or cone outer segments (ROS or COS) can be easily isolated in a dark room under dim red light (>650 nm) that will not activate and bleach the pigment (Figure 3). Further, in dark-adapted ROS, rhodopsin makes up about 85% of the total protein content (DeGrip et al., 1980). Eventually, dark-adapted bovine retinae even became commercially available (Hormel Co., Austin, Minnesota, United States). Finally, bovine rod rhodopsin was found to be relatively resistant to destabilization by detergents as compared to most other visual pigments, allowing extensive purification. Likewise, it proved to be sufficiently stable in less mild but more crystal-production-favoring small detergents like OG and NG to facilitate crystallization trials (Palczewski et al., 2000; Park et al., 2008).

The bovine rhodopsin amino acid sequence was established thanks to heroic protein sequencing efforts (Abdulaev et al., 1982; Hargrave et al., 1983). Over time, sequence information became available more easily via genome mining and c-DNA-sequencing. Thus, it came out that most invertebrate visual pigments are similar in size to the vertebrate pigments (36–42 kD), but mollusc pigments are significantly larger (46–55 kD), because of the presence of a much longer C-terminal (Ovchinnikov et al., 1988b; Gärtner, 2000). This additional stretch is unique in having an insertion of up to eleven copies of a peculiar pentapeptide sequence (Pro-Pro-Gln-Gly-Tyr), which probably helps in immobilization of the protein in the microvillar membrane (Ryba et al., 1993; Gärtner, 2000). Longer C-terminal stretches are also found in non-visual rhodopsins. For instance, the VA-opsin and melanopsin family also show this feature, except that the pentapeptide insertion does not occur. Here the extra sequence probably has a function in complex regulation of signal processing and desensitization (Valdez-Lopez et al., 2020; Contreras et al., 2021). Some VA-opsins and melanopsins are even produced in two or more splicing isoforms, with longer and shorter C-terminals (Davies et al., 2010).

While squid provides fair quantities of visual pigment, the first complete 3-D crystal structures only became available since 2008, both because of the much lower stability of the pigments in detergent solution and since crystallization could only be achieved after proteolytic removal of most of the long C-terminal (Murakami and Kouyama, 2008; Shimamura et al., 2008). The overall fold of the seven-transmembrane α-helical scaffold is quite similar to bovine rhodopsin, but the structure of the long C-terminal could not be determined, of course. The position of the retinal chromophore is slightly different, since the Glu residue functioning as the direct counterion for the protonated Schiff base is displaced from the site in the vertebrate pigments (Terakita et al., 2004). The first crystal structure of an arthropod rhodopsin (jumping spider) was only recently published in 2019, and again shows the familiar seven α-helical fold with overall high similarity with the squid structure (Varma et al., 2019). So far, crystal structures of non-visual rhodopsins have not been reported.

The crystal unit cell of bovine rod rhodopsin contains a dimer, but its interaction pattern is very different from the natural one (Fotiadis et al., 2006; Palczewski, 2006). In fact, rhodopsin is equally active as a monomer, and the organization in the ROS disc membranes is still debated (monomer, dimer, longer stretches?) (Fotiadis et al., 2004; Chabre and LeMaire, 2005; Mishra et al., 2016; Zhang et al., 2016; Feldman et al., 2019; Zhao et al., 2019). Invertebrate visual rhodopsins are probably rigidly immobilized in their native membrane, which allows to discern the polarization plane of the incoming light (Gärtner, 2000; Stavenga et al., 2000).

The crystal structures are essential to resolve the protein fold of the rhodopsins and have confirmed several conjectures of the binding pocket. Biochemical, vibrational (resonance Raman and FTIR spectroscopy) and solid-state NMR studies already produced very strong evidence that it indeed harboured the 11-*cis* configuration of retinal (Groenendijk et al., 1980; Mathies et al., 1987; Lugtenburg et al., 1988; DeGrip and Rothschild, 2000; Mathies and Lugtenburg, 2000). Surely enough, this configuration best fitted the non-protein electronic density in the binding pocket. The same is true for the covalent binding of retinal to a lysine residue, for which the above-mentioned techniques also already provided a wealth of evidence (Bownds, 1967; DeGrip et al., 1973; Creemers et al., 1999; Mathies and Lugtenburg, 2000). However, to firmly establish protonation of the Schiff base the resolution of the crystal structures is not high enough. Instead, the evidence produced by vibrational and NMR spectroscopy is very convincing and in fact was later underpinned by quantum-chemical computation (Palings et al., 1987; Herzfeld and Lansing, 2002; Gascón et al., 2005; Tastan et al., 2014).

### Type-1 Family

The sensitivity to detergent action also varies strongly between microbial rhodopsins. For instance, while bacteriorhodopsin (BR) is quite stable in OG, Triton X-100 and dodecylphosphocholine (DPC) even as a monomer, the rhodopsin proton pump from the cyanobacterium *Gloeobacter violaceus* (GR) strongly prefers DDM and is very unstable in DPC (Dencher and Heyn, 1978; Brouillette et al., 1989; Ganapathy et al., 2020). In general, OG and DDM are the preferred agents for solubilization of type-1 rhodopsins.

The spectral range of type-1 rhodopsins (360–690 nm) is comparable to that of type-2. There is less evidence for a clear relation to activity or habitat, an exception being the proton pump proteorhodopsin, which exhibits a blue-shift in deeper marine environments (Béjà et al., 2001; Bielawski et al., 2004). In a major distinction from type-2, microbial rhodopsins invariably exploit retinal A1 in the all-*trans* configuration as the basis for their light absorbance. Here, as well, a plethora of experimental evidence has demonstrated retinal binding to a lysine residue via a protonated Schiff base (Haupts et al., 1999).

Advanced angular electron diffraction studies on 2-D BR crystals in membrane patches already afforded a first glimpse into the organization of the helical transmembrane segments of type-1 rhodopsins (Henderson and Unwin, 1975; Mitra et al., 1993; Grigorieff et al., 1996; Heymann et al., 1997; Mitsuoka et al., 1999). The first 3-D crystal structures were reported for BR from 1997 onwards, and at a very high resolution slightly before that of bovine rhodopsin (Luecke et al., 1999; Pebay-Peyroula et al., 2000). This progress was aided by its high stability in detergent solutions and the relatively simple isolation from its native source. Bacteriorhodopsin is organized in large singular patches in the cellular membrane of *Halobacterium salinarum*, which can visually be observed and separated from other membrane fragments quite easily (Oesterhelt and Stoeckenius, 1971). In addition, type-1 rhodopsins complete a full photocycle (see below) and after photo-activation do not release the retinal, but thermally return to the ground state. This obviates the complexity of using dark rooms and shielding all experimental manipulations from room light exposure. Meanwhile, quite a number of crystal structures have been resolved for various classes of type-1 rhodopsins (Table 1). The most recent high resolution 3-D structures actually capitalized on the fantastic progress in cryo-EM (Hirschi et al., 2021; Kishi et al., 2022).

**TABLE 1:** Selected additional citations for the section “Spectral and structural properties and solubilization”.

*Type-1 pigments*
Optical spectroscopy: Rousso et al. (1998); Kanehara et al. (2017); Asido et al. (2021)
Vibrational spectroscopy: Garczarek and Gerwert, (2006); Lórenz-Fonfría and Kandori, (2009); Kraack et al. (2011); Verhoefen et al. (2011); Lórenz-Fonfría et al. (2015a); Ito et al. (2018); Watari et al. (2019); Lórenz-Fonfría et al. (2021)
NMR/EPR spectroscopy: Smith et al. (1989); Shi et al. (2009); Mao et al. (2014); Planchard et al. (2014); Shigeta et al. (2017); Mao et al. (2019); Naito et al. (2019); Friedrich et al. (2020)
Crystallography/EM: Havelka et al. (1995); Kimura et al. (1997); Belrhali et al. (1999); Subramaniam et al. (1999); Royant et al. (2001); Vogeley et al. (2004); Luecke et al. (2008); Wada et al. (2011); Kato et al. (2012); Wang et al. (2012); Frank et al. (2014); Kato et al. (2015); Nango et al. (2016); Tsukamoto et al. (2016); Broecker et al. (2017); Hasegawa et al. (2018); Ghanbarpour et al. (2019); Kovalev et al. (2019); Li et al. (2019); Morizumi et al. (2019); Shihoya et al. (2019); Yun et al. (2019); Besaw et al. (2020); Hayashi et al. (2020); Kovalev et al. (2020a); Lu et al. (2020); Bada Juarez et al. (2021); Higuchi et al. (2021); Li et al. (2021); Suzuki et al. (2022); Zhang et al. (2022)
Atomic force microscopy: Müller et al. (2002); Klyszejko et al. (2008); Yu et al. (2017); Heath et al. (2021)
Computational: Hayashi et al. (2001); Fujimoto et al. (2007); Melaccio et al. (2016); Karasuyama et al. (2018); Tsujimura and Ishikita, (2020); Fujimoto, (2021); Shen et al. (2021)
Reviews: Béjà et al. (2000); Caffrey, (2003); Engel and Gaub, (2008); Bamann et al. (2014); Grote et al. (2014); Neutze et al. (2015); Engelhard et al. (2018); Bibow, (2019); Kwon et al. (2020); Kawasaki et al. (2021)
Solubilization: Yu et al. (2000); Bayburt et al. (2006); Yeh et al. (2018); Ueta et al. (2020)
Other: Tribet et al. (1996)
*Type-2 pigments*
Optical spectroscopy: Seki et al. (1998); Salcedo et al. (1999); Schafer and Farrens, (2015); Katayama et al. (2019)
Vibrational spectroscopy: Rothschild et al. (1980); Kochendoerfer et al. (1999)
NMR/EPR spectroscopy: Creemers et al. (1999); Carravetta et al. (2004)
Crystallography/EM: Sardet et al. (1976); Davies et al. (1996); Davies et al. (2001); Krebs et al. (2003); Standfuss et al. (2007); Stenkamp, (2008); Hildebrand et al. (2009); Blankenship et al. (2015); García-Nafría and Tate, (2020); Zhang et al. (2021a)
Computational: Nikolaev et al. (2018); Patel et al. (2018)
Reviews: Neitz and Neitz, (1998); Spudich et al. (2000); Sakmar et al. (2002); McDermott, (2009); Smith, (2010); Bickelmann et al. (2015); Guo, (2020)
Solubilization: Kropf, (1982); Sadaf et al. (2015); Frauenfeld et al. (2016); Lee et al. (2020); Grime et al. (2021)

The available type-1 3-D structures show high similarity in protein fold and retinal pocket location. The basic seven α-helical transmembrane organization is comparable to that of type-2 (Figure 6), but for type-1 the helical packing is somewhat different and more compact. The loop segments connecting the helices are generally shorter and the retinal pocket is positioned differently to accommodate the longer all-*trans* chromophore instead of the curved 11-*cis* one (Figures 1, 6). Aspects of the binding pocket (retinal isomer and binding to a lysine residue via a Schiff base) again were in line with a wealth of evidence generated by biochemical and spectroscopic techniques (Lanyi, 2004). A recent XFEL study of the bacteriorhodopsin photocycle achieved a very high structural (ca 1.5 Å) and temporal (femtosecond) resolution and produced evidence for protonation of the Schiff base (Nogly et al., 2018). Also in the type-1 case the evidence generated by biophysical techniques like vibrational, EPR and NMR spectroscopy and by quantum-chemical computation is most convincing (Ernst et al., 2014; Brown and Ernst, 2017; Ryazantsev et al., 2019; Nagata and Inoue, 2022).

A conspicuous feature of most type-1 rhodopsins is that they organize in homo-oligomers, whether observed in the native membrane or in host cells. The most common arrangement for bacterial and archaeal rhodopsins are trimers or pentamers, though occasionally hexamers do occur as well (Hussain et al., 2015; Shibata et al., 2018; Kao et al., 2019). Circular dichroism spectroscopy provides evidence for exciton coupling between the chromophores (Cassim, 1992; Fujimoto and Inoue, 2020; Fujimoto, 2021). For eukaryotic type-1 rhodopsins, homo-dimeric as well as hetero-dimeric complexes are observed (Mukherjee et al., 2019; Govorunova et al., 2021; Broser, 2022). Isolated type-1 monomers are also functionally active, indicating that the oligomeric assembly probably affords optimal packing and mutual stabilization, and/or the opportunity to modulate monomer activity by inter-subunit interplay (Iizuka et al., 2019).

A novel feature was discovered in the enzyme-rhodopsins i.e. an additional transmembrane segment at the N-terminal (TM8), which functions as a connector with the cognate soluble enzyme domain and seems to be essential for modulating its activity (Ikuta et al., 2020; Tsunoda et al., 2021).

Interestingly, several thermostable microbial rhodopsins have been discovered. The crystal structure of the highly thermophilic rhodopsin (TR) from *Thermus thermophilus* was resolved to be very similar to that of the much less thermally stable xanthorhodopsin (XR) from *Salinibacter ruber*, including the binding crevice for the carotenoid antenna (Tsukamoto et al., 2016). Likewise, the crystal structure of the thermostable rhodopsin proton-pump from *Rubrobacter xylanophilus* (RxR) is very similar to that of bacteriorhodopsin (Hayashi et al., 2020). An unusually widely stable proton pump (pH, detergent, temperature), named Tara76 rhodopsin, was isolated from uncultured bacteria (Shim et al., 2021). Such data shed new light on the design options to increase thermal and environmental stability without a significant sacrifice in dynamics and activity (Hayashi et al., 2020).

Additional selected references relevant for this section have been compiled in Table 1.

## Functional Diversity, Phylogeny

It was relatively simple in the old days. On one hand, we knew of animal rhodopsins, being G protein-coupled receptors, very nicely developed and evolved into a set of proteins allowing photopic vision (color discrimination) and a single class for extremely sensitive scotopic vision (black-and-white). On the other hand, another class of retinal-proteins had evolved in archaea to exploit solar energy for active transport of protons and chloride ions. However, with the awakening of the genome era, this view became totally obsolete. While the notion that the type-1 and type-2 families probably do not have a common ancestor and have little overlap in physiological function was consistent, over time many new members were discovered and their classification revised (Porter et al., 2012; Yee et al., 2013; Zabelskii et al., 2021). In hindsight, it was to be expected that ahead of the large carotenoid and chlorophyll dependent protein complexes in the photosynthetic reaction centers, Nature would have taken advantage of the abundance of solar energy making maximal use of this fantastic toolbox of retinal-proteins, that are relatively simply to bioproduce and adapt.

It is likely that many products of this toolbox are yet to be discovered, but already the genetic and functional diversity is so vast and complex, that we provide a very general overview below and mostly refer to selected reviews.

### Type-2 Family

The animal rhodopsins have meanwhile been classified in at least nine gene families (Opn1–Opn9) and two separate sets with some members still awaiting further assignment (Table 2). Physiological function and tissue distribution show incredible diversity (Janssen et al., 2003; Leung and Montell, 2017; Liebert et al., 2021; Moraes et al., 2021; Calligaro et al., 2022). The classical visual pigments come within Opn1 (cone pigments) and Opn2 (rod pigments). Pigments discovered later in the vertebrate retina, such as melanopsin, VA-opsin or neuropsin, peropsin (RRH) and RGR fall under Opn4, unclassified and Opn5, respectively (Table 2). The common thread still is primary signal transduction via at least one of the available G-protein species (Gt, Go, Gi, Gq, and Gs), with cross-activation, modulation or desensitization via a variety of other mediators. However, RGR and its mollusc counterpart retinochrome are exceptional in this context, since they act as photo-isomerases, binding all-*trans* retinal in the dark state, and releasing 11-*cis* retinal after photo-activation as a supply for regeneration of visual opsins (Hara et al., 1967; Pepe and Cugnoli, 1992; Zhang et al., 2019; Choi et al., 2021; Vöcking et al., 2021). Another remarkable subset are Opn5L, peropsin and Opn7 members, which also bind all-*trans* retinal in the dark state, but that seems to be the active state binding the G protein. Upon illumination they generate the 11-*cis* chromophore, which represents the resting state that in the case of Opn5L members may even thermally revert to the active state (Nagata et al., 2018; Yamashita, 2020; Karapinar et al., 2021; Sakai et al., 2022). An even more surprising observation is that some type-2 pigments may be involved in recognizing temperature differences or mechanical changes, or function as chemosensors or tumorigenic elements, possibly even without requiring their retinal cofactor (Shen et al., 2011; Park et al., 2013; Baker et al., 2015; Pérez-Cerezales et al., 2015; Leung et al., 2020; Xu et al., 2020; Córdova et al., 2021; Moraes et al., 2021).

**TABLE 2:** Current classification of type-2 rhodopsins.

Gene family or group	Main compo-nents^b^	Spectral range^a^	Location	Mono/bi-stable^a^	Special facts	Selected literature
Opn1	Vertebrate cone pigments	350–610 nm	Retina	Mono		Nathans, (1987); Imamoto and Shichida, (2014); Hofmann and Palczewski, (2015); Borgia et al. (2018); Jacobs, (2018); El Khatib and Atamian, (2019); Astakhova et al. (2021)
Opn2	Vertebrate Rod pigments	440–520 nm	Retina, Brain	Mono	Includes exorhodopsin	Ebrey and Koutalos, (2001); Rohrer et al. (2003); Warrant and Locket, (2004); Tarttelin et al. (2011); Davies et al. (2012); Liu et al. (2019); Ortega and Jastrzebska, (2019)
Opn3	Encephalopsins	Blue-green	Multiple tissues, Extra-ocular	Bi		Blackshaw and Snyder, (1999); Halford et al. (2001); Moutsaki et al. (2003); Leung and Montell, (2017); Lan et al. (2020); Olinski et al. (2020); Xu et al. (2020); Davies et al. (2012); Davies et al. (2021); Liebert et al. (2021)
	Panopsins					
	TMT-opsins					
Opn4	Melanopsins	450–500 nm	Multiple tissues	Bi	Long C-terminals	Provencio et al. (1998); Provencio et al. (2000); Panda et al. (2002); Kumbalasiri and Provencio, (2005); Panda et al. (2005); Giesbers et al. (2008); Davies et al. (2010); Shirzad-Wasei and DeGrip, (2016); Duda et al. (2020); Valdez-Lopez et al. (2020); Contreras et al. (2021)
Opn5**^c^**	Neuropsins	UV-blue	Multiple tissues	Bi	11-*cis* - > all-*trans*	Jiang et al. (1993); Sun et al. (1997); Tarttelin et al. (2003); Yamashita et al. (2010); Yamashita et al. (2014); Nagata et al. (2018); Sato et al. (2018b); Zhang et al. (2019); Yamashita, (2020); Choi et al. (2021); Liu et al. (2021); Calligaro et al. (2022); Fujiyabu et al. (2022)
	Peropsins				Photoactivation	
	RGR’s				All-*trans* -> 11-*cis*	
Opn6		UV-blue	Multiple tissues	Mono and Bi	Zebrafish	Davies et al. (2015)
					Monotrenes	
Opn7		UV-blue	Multiple tissues	Bi and Mono	Zebrafish	Davies et al. (2015); Karapinar et al. (2021)
					All-*trans* -> 11-*cis*	
Opn8		UV-blue	Multiple tissues	Bi	Not in mammals	Davies et al. (2015)
Opn9		?	Multiple tissues	?	Zebrafish, long extra-cellular loop	Davies et al. (2015)
R (habdomeric) opsins	Molluscs	340–600 nm	Mainly ocular	Bi	Molluscs, long C-terminal	Wald, (1953); Hara et al. (1967); Hillman et al. (1983); Vogt and Kirschfeld, (1984); Gärtner, (2000); Stavenga et al. (2000); Furutani et al. (2005); Porter et al. (2012); Nagata et al. (2018); Leung et al. (2020); Nagata and Inoue, (2022)
	Arthropods					
Cn(iderian) opsins	Jellyfish	?	Multiple tissues	?		Musio et al. (2001); Plachetzki et al. (2012); Porter et al. (2012); Feuda et al. (2014); Davies et al. (2015); Gerrard et al. (2018); Hayashi et al. (2020)
Separate gene groups	VA-opsins	Blue-green	Multiple tissues	Mostly Bi	Parietopsins mainly in pineal gland	Okano et al. (1994); Blackshaw and Snyder, (1997); Kojima et al. (1997); Soni and Foster, (1997); Nakamura et al. (1999); Spudich et al. (2000); Foster and Hankins, (2002); Su et al. (2006); Davies et al. (2010); Tsukamoto and Terakita, (2010); Passananeck et al. (2011); Sakai et al. (2012); Koyanagi et al. (2014); Davies et al. (2015); Leung and Montell, (2017); Sato et al. (2018a); Pérez et al. (2019b); Rawlinson et al. (2019); Döring et al. (2020); Eickelbeck et al. (2020); Copits et al. (2021); Rodgers et al. (2021)
	Parapinopsins				Xenopsins and Go-rhodopsins in invertebrates	
	Parietopsins					
	Pinopsins					
	Xenopsins					
	Go-rhodopsins					

a Spectral range and mono/bistability not always exclusive within a group and very limited known for Opn6-Opn9 and Cn-opsins.

b Cone pigments are mainly involved in color (photopic) vision, rod pigments in (scotopic) dim-light vision. In mammals melanopsins are important for pupillary contraction and circadian regulation. Retinochromes (R-opsins) and peropsins and RGRs (Opn5) have photoisomerase activity (all-trans → 11-cis).

c The Opn5L group (Sato et al., 2018b; Yamashita, 2020) may have been classified wrongly, since they clade within the Opn6-9 framework.

### Type-1 Family

**FIGURE 7:**
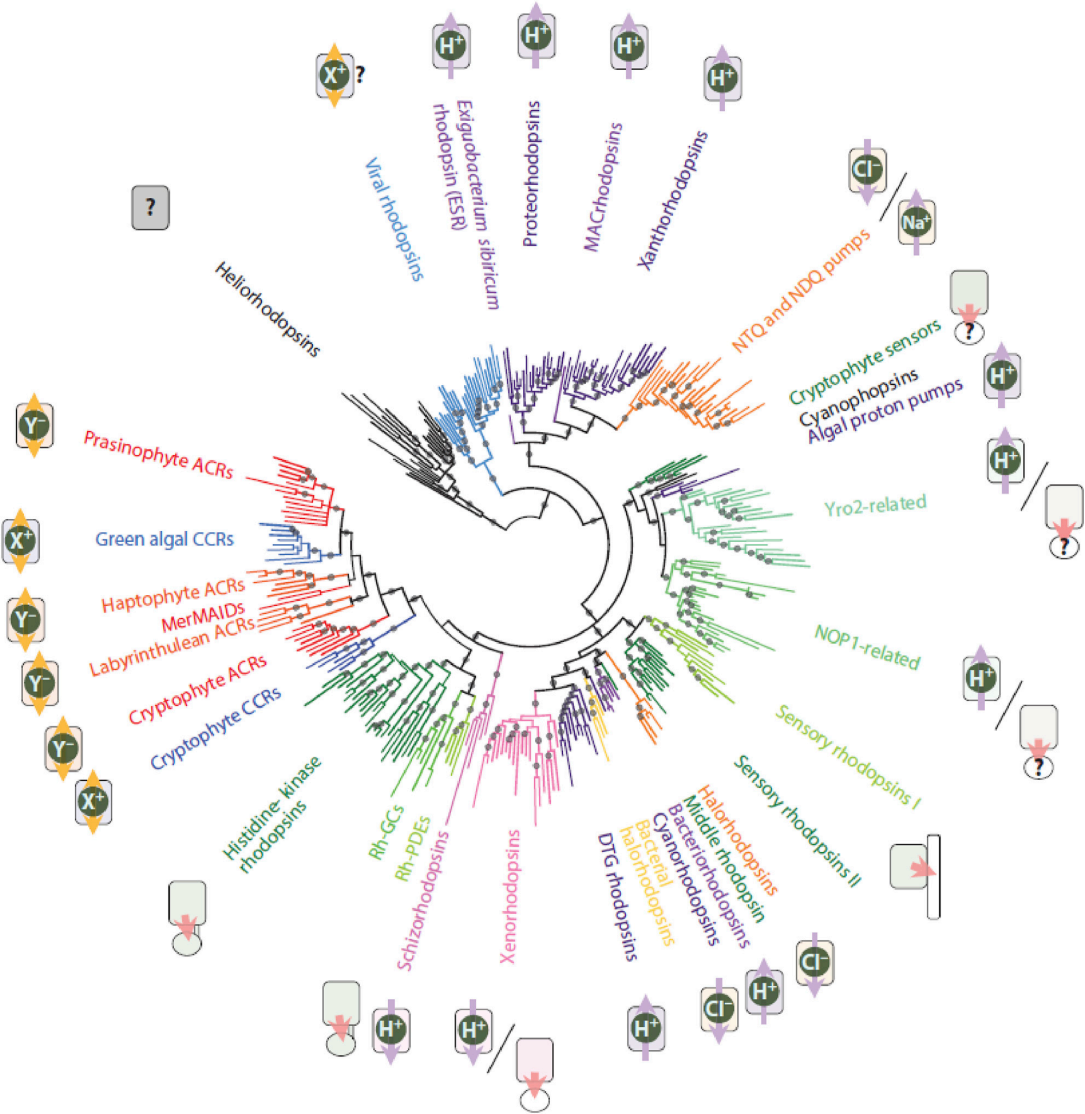
Global phylogeny of type-1 pigments illustrating their formidable diversification. The figure was modified with consent from Rozenberg et al., 2021. We refer to the original paper for the construction of the tree and for all abbreviations. Purple arrows represent active outward (away from center) and inward ion transport, respectively. Orange arrows represent ion channels. Pink arrows represent enzyme-rhodopsins (fused enzyme domains) and sensory rhodopsins (detachable transducers). For further details of the various classes we refer to recent literature (Hasemi et al., 2016; Govorunova et al., 2016; id-, 2022; Nakajima et al., 2018; Oppermann et al., 2019; Kovalev et al., 2020a; Rozenberg et al., 2021).

Type-1 rhodopsins have been identified in archaea and eubacteria, including cyanobacteria, as well as in unicellular eukaryotes (algae, fungi, yeast) and more recently also in choanoflagellates and viruses (Lamarche et al., 2017; Bratanov et al., 2019; Zabelskii et al., 2020; Rozenberg et al., 2021; Govorunova et al., 2022b; Nagata and Inoue, 2022). Most of these pigments function as light-driven ion transporters or ion channels (Figure 7). The newly discovered xenorhodopsins and schizorhodopsins are exceptional as they perform inward-directed proton transport (Inoue et al., 2018; Inoue et al., 2020; Weissbecker et al., 2021; Brown, 2022). However, some type-1 rhodopsins display a photosensory function (sensory rhodopsins) and signal via a cognate transducer protein, which is totally different functionally and structurally from the animal G-proteins (Bogomolni and Spudich, 1991; Krah et al., 1994; Deininger et al., 1995). Overall, type-1 pigments are the dominant contributors to marine phototrophy (Casey et al., 2017; Larkum et al., 2018; Gómez-Consarnau et al., 2019). In addition, eukaryotic type-1 rhodopsins have been discovered which are intracellularly fused to an enzymatic domain and mediate light-driven enzyme activation (guanylyl cyclase, phosphodiesterase) or inhibition (guanylyl cyclase, based upon histidine kinase activity) (Avelar et al., 2014; Lamarche et al., 2017; Luck et al., 2019; Mukherjee et al., 2019; Tsunoda et al., 2021; Broser, 2022; Tian et al., 2022). These pigments have been termed as enzyme-rhodopsins.

The overall structure and photochemistry of all type-1 rhodopsins present a very similar pattern, though the sequence identity can be as low as 12%, and the kinetics of the photocycle can vary up to at least thousand-fold. The most recent addition, the heliorhodopsins, are not very different in their protein fold from e.g. BR in spite of a very low sequence identity (<10%) (Shihoya et al., 2019). Considering their inverted insertion into the membrane, very long photocycle and so far unknown functionality, they probably are better classified separately as type-3 rhodopsins (Tanaka et al., 2020; Chazan et al., 2022).

## Heterologous Expression and Purification

The congruent broad heterogeneity in the rhodopsin superfamily offers a fascinating spectrum for mechanistic studies as well as biomimetic adaptation and application. However, mechanistic studies still require large quantities of relatively pure material (at least several mg). With the exception of some visual pigments and archaeal rhodopsins, such quantities are not available from native sources. Besides, purifying minor quantities of rhodopsins out of a large excess of cellular membrane proteins turned out to be a “hell of a job” (Dartnall, 1962b; Hubbard et al., 1971). Furthermore, in-depth mechanistic studies and biomimetic applications need the ability to make modifications biosynthetically at the protein residue level, and synthetically at the chromophore level. And even when *in silico* molecular dynamics and quantum chemical computation would have reached the time-scale of protein conformational changes (femtoseconds to seconds range) and the native accuracy, then still experimental verification is in order. Experimentally modifying rhodopsins in the native organism was completely out of hand at the time, except for some limited success with bacteriorhodopsin mutants in *Halobacterium salinarum* which still did not solve the quantity requirement (Krebs et al., 1993). Hence, the search for suitable heterologous expression hosts started in the 1980s, and over time it became obvious that the eukaryotic rhodopsins required quite a different perspective.

With the start of the genome era, recombinant DNA technology (genome mining, DNA and c-DNA sequence information and comparison, DNA sequence modification) became accessible and have now become common experimental tools (Khorana, 1979; Khorana et al., 1987). Likewise, total synthesis of retinal isomers and a plethora of derivatives has improved significantly (Dawadi and Lugtenburg, 2010; Liu and Liu, 2011; Álvarez et al., 2014; El-Tahawy et al., 2020).

### Type-2 Family

Type-2 rhodopsins can undergo a variety of posttranslational modifications (disulfide-bridge formation, N- and O-glycosylation, methylation, acetylation, myristylation, palmitoylation, phosphorylation), most of which are not properly executed by the bacterial or archaeal biosynthetic machinery (Table 3). Expression of bovine rhodopsin in bacteria and even yeast did not yield promising results (Mollaaghababa et al., 1996; Abdulaev and Ridge, 2000). Hence, for optimal heterologous expression a eukaryotic cell type had to be selected as a host. Attempts have been made to express type-2 pigments and related receptors in the eye of whole organisms (mouse, Xenopus) and in *Caenorhabditis elegans* using viral vectors or transgenic animals*,* but this gave relatively low yields or even led to retinal degeneration (Zhang et al., 2005; Salom et al., 2008; Cao et al., 2012; Salom et al., 2012). Eventually, the best results with sufficient posttranslational modification and targeting to the plasma membrane were obtained in some mammalian cell lines using plasmid transfection (COS, HEK, Neuroblastoma cell lines), in insect cell lines using baculoviral infection (Spodoptera Sf9 and Sf12 and Trichoplusia “High-Five”) and in Xenopus oocytes (Oprian et al., 1987; Janssen et al., 1988; Khorana et al., 1988; Karnik et al., 1993; Kazmi et al., 1996). The highest expression levels of functional pigments, with addition of 11-*cis* retinal during culture or after isolation of the cells, were obtained in suspension culture of insect cells or specially adapted HEK293 cells, with yields up to 130 nmol/L, equivalent to ca 5 mg bovine rhodopsin per liter (Klaassen and DeGrip, 2000; Reeves et al., 2002). Even then the pigment accounts for maximally 5 percent of the total cellular membrane protein, and further purification is inevitable. Eventually, gene manipulation lent a helping hand and it has now become common practice to add a small sequence tag to the pigment c-DNA, encoding a short peptide sequence to easily identify and purify the expressed pigment. Two approaches have become the most popular in the type-2 rhodopsin field. One exploited the availability of a monoclonal antibody against the C-terminal octapeptide of bovine rhodopsin (Molday, 1989). This allows for highly selective immuno-affinity purification using a suitable detergent like DDM for solubilization (Ridge et al., 1995). By adding to or replacing the native C-terminal with this octapeptide, the resulting tagged protein can be comfortably isolated. The second approach involved extending the C-terminal with six to ten histidine residues (His6-tag to His10-tag), which upon solubilization with a suitable detergent allows metal affinity purification over a matrix containing immobilized Ni^2+^ or Co^2+^ complexes (Janknecht et al., 1991; Janssen et al., 1995). Both approaches are very effective with hardly any perturbation of expression level and functionality of the pigment (Reeves et al., 1999; Bosman et al., 2003). Nevertheless, if necessary, a short target peptide sequence for a selective proteolytic enzyme can be introduced in front of the purification tag to remove it after purification (Sarramegna et al., 2006). Most Opn1 and Opn2 pigments can be satisfactorily purified by either procedure (Vissers and DeGrip, 1996; Shirzad-Wasei and DeGrip, 2016; Katayama et al., 2017; Katayama et al., 2019). Some pigments from the other subsets have been difficult to solubilize or are too unstable in detergent solution to survive purification. The alternative option then is to transfer the protein into the stabilizing lipid environment of nanodiscs (Figure 4), which requires hardly any detergent (amphipol or SMA-type) or very brief exposure to a suitable mild detergent (MSP-type). Exploiting the sequence tag on the incorporated protein, the protein-nanodisc unit is then easily purified again by affinity chromatography (Shirzad-Wasei et al., 2015; Cai et al., 2017; Ganapathy et al., 2020).

**TABLE 3:** Selected additional citations for the section “Heterelogous expression and purification”.

*Type-1 pigments*
Optical spectroscopy: Chen and Gouaux, (1996); Kwon et al. (2019)
Posttranslational: Hildebrandt et al. (1991); Müller, (1992); Lang-Hinrichs et al. (1994); Feng et al. (2013)
Review: LinCereghino and Cregg, (2000); Lichty et al. (2005); Hasegawa et al. (2020)
Other: Schey et al. (1992)
*Type-2 pigments*
Optical spectroscopy: Oprian et al. (1991); Kojima et al. (1995); Radlwimmer and Yokoyama, (1997); Ma et al. (2001); Melyan et al. (2005); Qiu et al. (2005); Giesbers et al. (2008); Shirzad-Wasei et al. (2013); Kahremany et al. (2019)
Vibrational spectroscopy: Katayama et al. (2012); Katayama et al. (2017)
Posttranslational: Hargrave, (1977); Karnik et al. (1988); Ovchinnikov et al. (1988a); Janssen et al. (1991); O'Tousa, (1992); Fujita et al. (1994); Kaushal et al. (1994); Morello and Bouvier, (1996); Nakagawa et al. (1997); Zhang et al. (1997); Katanosaka et al. (1998); Gibson et al. (1999); Hwa et al. (1999); Ridge and Abdulaev, (2000); Maeda et al. (2003); Park et al. (2009); Tam and Moritz, (2009); Salom et al. (2019)
Expression: Schey et al. (1992); Harada et al. (1994); Townson et al. (1998); Reeves et al. (2002); Peirson et al. (2004); Panda et al. (2005)
Review: Hargrave, (1982)

The opportunity to modify, bio-generate and purify type-2 pigments in sufficient quantities has given a tremendous boost to all mechanistic and functional studies. Analysis of the native proteins or binding pocket mutants, often in combination with ^2^H-, ^13^C- or F-labeling and/or chemical modification of retinal and/or with ^15^N- and/or ^13^C-labeling of protein residues or inserting modified amino acids, has provided a wealth of data, underpinning, extending and refining the information obtained from crystal structures (see next sections). Groundbreaking details of dark state structures have been excavated by biochemical (e.g. limited proteolysis, selective chemical modification, selective deuteration, atomic force microscopy, cryo-EM) and biophysical studies (e.g. FTIR and resonance Raman spectroscopy, solid-state NMR spectroscopy, EPR spectroscopy) (Table 3). This also fueled a large body of theoretical and *in-silico* efforts (molecular dynamics, quantum-chemical calculation and modeling) (Ryazantsev et al., 2019; Pedraza-González et al., 2020; Fujimoto, 2021; Mroginski et al., 2021; Church et al., 2022b). As a result of all these exertions, it has already been possible to construct a highly detailed picture of the dark state of bovine rhodopsin.

### Type-1 Family

For the archaeal and bacterial type-1 rhodopsins, a heterologous expression host was more easily identified. *Escherichia coli* strains had already been developed for uncomplicated suspension culture, high productivity, low proteolytic activity and easy transformation. Plasmids with inducible promoters became available, and were further engineered with specific features, like producing the necessary enzymatic machinery to generate all-*trans* retinal from its precursor β-carotene (Kim et al., 2008). Nevertheless, in most cases just supplementing the cell culture with all-*trans* retinal together with inducing opsin expression or even after membrane isolation was sufficient to produce the full equivalent of the corresponding rhodopsin (Spudich et al., 2000; Ganapathy et al., 2015). In this way yields up to 20 mg/L have been reported (Ganapathy et al., 2015; Song et al., 2020). For some archaeal pigments, this straightforward approach only gave low yields and had to be adapted e.g. for bacteriorhodopsin itself (Bratanov et al., 2015; Tu et al., 2018). On the other hand, heterologous expression was more problematic for the eukaryotic type-1 rhodopsins, again because of their more complex posttranslational modification profile. Channelrhodopsins are commonly produced in yeast (*Pichia pistoris*), but successful production of eukaryotic type-1 pigments in insect and mammalian cell lines, *Caenorhabditis elegans* and Xenopus oocytes is also reported (Nagel et al., 2003; Bruun et al., 2015; Govorunova et al., 2017). An interesting new approach is using the trypanosome *Leishmania tarentolae* for over-expression (Volkov et al., 2017). For optogenetic applications (see below), functional production and targeting in a mammalian context is imperative, and often requires insertion of trafficking or targeting signals and/or sequence optimization to mammalian genetic code preferences.

The C-terminal His-tag has become the most popular option for purification of archaeal and eubacterial rhodopsins. For eukaryotic type-1 rhodopsins, several tags are used, including the His-tag, although the latter may sometimes interfere with particular electrophysiological or enzymatic analyses (Govorunova et al., 2021; Rozenberg et al., 2021; Tsunoda et al., 2021; Govorunova et al., 2022b).

Thanks to the powerful combination of the recombinant DNA toolbox with heterologous expression and purification making sufficient protein material available, an astounding repertoire of structural and functional data has also become available for the type-1 rhodopsins (Table 3). As a result, bacteriorhodopsin has become the best studied and fathomed membrane protein, with unprecedented insight into its structure and function (Ernst et al., 2014; Larkum et al., 2018; Nogly et al., 2018; Weinert et al., 2019). Next to that, the type-1 community has delivered prospects for a wealth of biotechnological and biomimical applications, far beyond any prognosis (see below).

## Photochemical Properties

**FIGURE 8:**
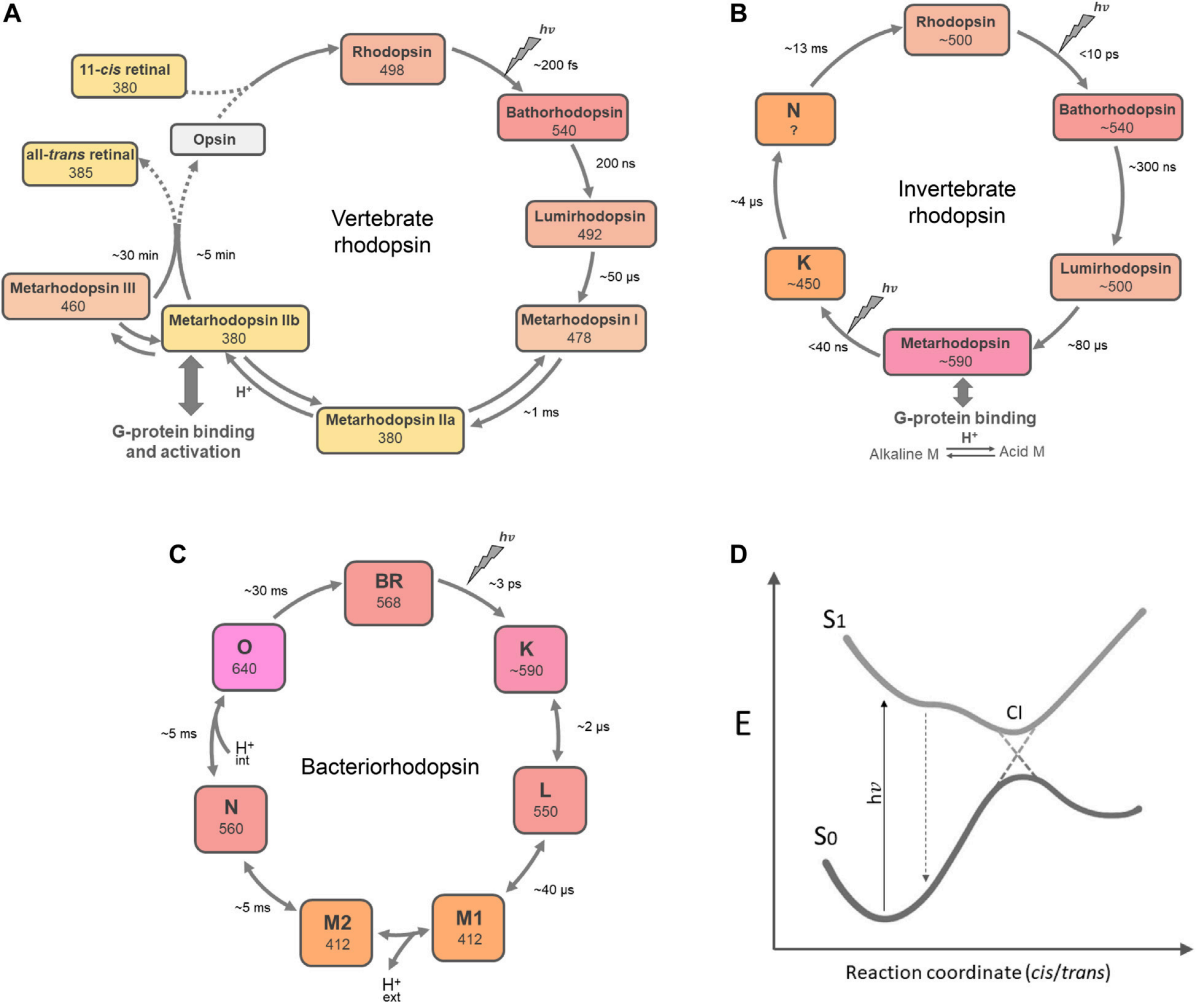
Global presentation of the predominant photochemical pathways in the rhodopsin families. **(A)** Bovine rod rhodopsin as the archetype of the monostable type-2 pigments, **(B)** squid/fly visual pigment chimera, typical for the bistable type-2 pigments, and **(C)** bacteriorhodopsin (BR) as a prototype for the type-1 pigments. The “dark state” 11-*cis*, 15-*anti* chromophore configuration in type-2 pigments is photo-excited into all-*trans.* The all-*trans* chromophore configuration in type-1 pigments is photo-excited into 13-*cis*, 15-*anti*, which thermally relaxes, eventually returning to the ground state. The early photo-intermediates still contain a protonated Schiff base and relax thermally to deprotonated Meta II or M states. In proton pumps like BR this is accompanied by opening up proton pathways in the protein, while in most type-2 pigments binding of a G protein is initiated. At this stage, the pathways divert, as further explained in the text. Of course, here are exceptions: some type-2 pigments contain all-*trans* in the “dark state,” which is photoexcited into 11-*cis*, either to change activity or to release 11-*cis* retinal for regeneration of visual opsins (Table 2). Some type-1 pigments can also photo-generate 9-*cis* or 11-*cis* states with deviating photocycles and/or functions. **(D)** Simplified schematic of a conical intersection where the excited chromophore at the S1 energy surface can cross over to the S0 energy surface of the photoproduct. The S1 surface can contain thermal transitions, and in type-1 pigments the kinetics to reach and cross-over at the conical intersection also depend on the pKa of the direct counterion to the Schiff base (Chang et al., 2022).

The initial rapid steps after photoactivation of type-1 and type-2 rhodopsins are quite comparable (Figure 8). Ultrafast photoisomerization of the chromophore leads to the first stable photoproduct within ps. This conversion is extremely efficient with quantum yields between 0.6 and 0.7 for type-2 pigments and varying between 0.3 and 0.7 for type-1 pigments and very low energy loss through fluorescence (Gozem et al., 2017). Often, this red-shifted photoproduct then thermally relaxes via spectrally distinguishable photo-intermediates within ms to a blue-shifted M(eta) intermediate, where the chromophore-binding Schiff base has become deprotonated through transfer of the proton to the direct counterion (Nakagawa et al., 1999; Hofmann, 2000; Tsukamoto and Terakita, 2010; Ernst et al., 2014; Govorunova et al., 2017). This explains the large blue-shift. In some type-1 pigments, a deprotonated M state is not formed, however a protonated L-like equivalent is observed (Spudich et al., 2014; Govorunova et al., 2017; Engelhard et al., 2018). The M or its L-like equivalent intermediate is the active state of the pigment, where the conformational changes in the protein evoke the subsequent cognate activity (grouping with cognate G protein or transducer, opening up an ion channel or vectorial ion pathway, regulating the enzymatic domain, etc.) (Table 4). At the M or L-like stage the type-2 and type-1 pathways take completely different directions.

**TABLE 4:** Selected additional citations for the section “Photochemical properties”.

*Type-1 pigments*
Optical spectroscopy: Butt, (1990); Ogonah et al. (1991); Chizhov et al. (1996); Inoue et al. (2004); Rupenyan et al. (2008); (2009); Inoue et al. (2011); Bayraktar et al. (2012); Ogren et al. (2015); Tahara et al. (2015); Iyer et al. (2016); Hontani et al. (2017a); Hontani et al. (2017b); Smitienko et al. (2017); Inoue et al. (2018); Chang et al. (2019); Kao et al. (2019); Luck et al. (2019); Tahara et al. (2019b); Hontani et al. (2020); Smitienko et al. (2021); Sugimoto et al. (2021); Chang et al. (2022)
Vibrational spectroscopy: Rothschild et al. (1981); Rothschild and Marrero, (1982); Rothschild et al. (1984); Marrero and Rothschild, (1987); Rödig et al. (1999); McCamant et al. (2005); Amsden et al. (2007); Neumann et al. (2008); Schäfer et al. (2009); Sasaki et al. (2011); Sudo et al. (2011); Saint Clair et al. (2012a); Johnson et al. (2014); Liebel et al. (2014); Kuhne et al. (2015); Lórenz-Fonfría et al. (2015b); Schnedermann et al. (2016); Yi et al. (2017); Roy et al. (2018); Kataoka et al. (2019); Kuhne et al. (2019); Kaufmann et al. (2020); Fischer et al. (2021); Polito et al. (2021)
NMR/EPR spectroscopy: Hu et al. (1998); Ding et al. (2018)
Crystallography/EM: Schobert et al. (2002); Frank et al. (2014); Furuse et al. (2015); Kato et al. (2015a); Wickstrand et al. (2015); Hosaka et al. (2016); Ikuta et al. (2020); Kojima et al. (2020c); Kovalev et al. (2020a); Bada Juarez et al. (2021); Hirschi et al. (2021); Li et al. (2021); Axford et al. (2022); Kishi et al. (2022); Poddar et al. (2022)
Computational: Schapiro and Ruhman, (2014); Feng and Mertz, (2015); Yalouz et al. (2021)
Review: Wand et al. (2013); Kandori et al. (2018); Buhrke and Hildebrandt, (2020)
*Type-2 pigments*
Optical spectroscopy: Yoshizawa and Wald, (1967); Regan et al. (1978); Shichida, (1986); Imamoto et al. (1989); Lewis et al. (1990); Gärtner et al. (1991); Davidson et al. (1994); Imai et al. (1995); Imamoto et al. (1996); DeLange et al. (1997); Jäger et al. (1997); Lewis et al. (1997); Vought et al. (1999); Kusnetzow et al. (2001); Furutani et al. (2003); Sato et al. (2011); Tarttelin et al. (2011); Gulati et al. (2017); Van Eps et al. (2017); Nagata et al. (2019); Chawla et al. (2021); Sakai et al. (2022)
Vibrational spectroscopy: Rothschild et al. (1976); Rothschild et al. (1983); DeGrip et al. (1985); Pande et al. (1987); DeGrip et al. (1988); Bagley et al. (1989); Masuda et al. (1993); Rath et al. (1993); Hashimoto et al. (1996); Rath et al. (1998); DeLange et al. (1999); Ritter et al. (2004); Yan et al. (2004); Ye et al. (2010); Nonaka et al. (2020); Hanai et al. (2021)
NMR/EPR spectroscopy: Smith et al. (1992); Verhoeven et al. (2001); Struts et al. (2007); Altenbach et al. (2008); Eilers et al. (2012); Brinkmann et al. (2018)
Crystallography/EM: Ruprecht et al. (2004); Schertler, (2005); Nakamichi and Okada, (2006); Scheerer et al. (2008); Choe et al. (2011); Murakami and Kouyama, (2011); (2015); Panneels et al. (2015); Tsai et al. (2019)
Atomic force microscopy: Kawamura et al. (2013)
Computational: Schreiber et al. (2006); Bhattacharya et al. (2008); Tavanti and Tozzini, (2014); Feng et al. (2015); Ren et al. (2016); Tomobe et al. (2017); Demoulin et al. (2021)
Review: Zundel, (1988); Yoshizawa and Kandori, (1991); Farrens, (2010); Smith, (2010); Polli et al. (2015); Vlasov et al. (2020)
Other: Angel et al. (2009); Bayburt et al. (2011)

### Type-2 Family

For the type-2 rhodopsins ultrarapid spectroscopy data are limited, and mainly available for Opn1, Opn2, and R-type pigments (Shichida et al., 1978; Shichida, 1990; Schoenlein et al., 1991; Vought et al., 2000; Imamoto and Shichida, 2014; Schnedermann et al., 2018). Generally speaking, two schemes have been identified: Monostable pigments eventually release all-*trans* retinal (all Opn1 and Opn2 rhodopsins, Figure 8A) following which the opsins require supplementation with retinal re-isomerized elsewhere to regenerate the original “dark” state. Bistable pigments (most other type-2 pigments investigated, Table 2) progress until a stable M-intermediate is reached (all-*trans* chromophore), that requires photo-isomerization to return to the original “dark” state (11-*cis* chromophore) (Figure 8B) (Hillman et al., 1983; Gärtner, 2000; Stavenga et al., 2000).

The photochemical profile of the monostable bovine rod rhodopsin has been explored in great detail. The native pigment and a variety of isotopically labeled and/or mutant pigments have been investigated by femtosecond optical spectroscopy and vibrational and NMR spectroscopy. These studies have revealed intimate details on the kinetics, conformational changes in the chromophore and surrounding H-bonded networks with constrained water molecules, protein-chromophore interplay and Schiff base (de)protonation (Table 4). Overall protein conformational changes have been elucidated by fluorescence, ESR and NMR spectroscopy and TR-WAXS (DeGrip et al., 1999; Kusnetzow et al., 2006; Alexiev and Farrens, 2014; Malmerberg et al., 2015; Van Eps et al., 2017; Smith, 2021). Crystal structures have been resolved for all photo-intermediates and present a broad structural basis (Table 4). The power of theoretical and quantum-chemical calculations has grown immensely, laying a strong foundation for electronic and energetic elements of the process, in particular (Schapiro et al., 2011; Gozem et al., 2017; Schnedermann et al., 2018; Agathangelou et al., 2021; Nikolaev et al., 2021).

A very effective combination of selectively labeled chromophore with femtosecond spectroscopy and advanced quantum chemical computation resolved many remaining issues in the photoisomerization process of bovine rhodopsin (Schnedermann et al., 2018). The global picture has arisen that after photo-excitation of the chromophore into the Franck-Condon state it rapidly relaxes along a barrierless trajectory on the potential surface to a minimal energy conical intersection (Figure 8D). Here, productive resonance of the electronic wave packet at the excited state potential surface with torsional and HOOP vibrational modes in the twisted C10-C13 segment of the 11-*cis* chromophore, can prime very effective cross-over to a ground state energy surface, generating a hot all-*trans*oid state (photorhodopsin) within tens of fs (Johnson et al., 2015). This relaxes thermally in about 200 fs into the photoproduct bathorhodopsin, which contains a still highly twisted all-*trans* chromophore, but is stable below 130 K (Yoshizawa and Wald, 1963). In free retinal, photoexcitation results in formation of several isomers (predominantly all-*trans*, 13-*cis*, 9-*cis*, and 11-*cis*), but in rhodopsins this conversion is remarkably selective from 11-*cis* to all-*trans.* This is clearly facilitated by the constraints of the binding site and the twist in the C10-C13 segment of the chromophore (Bismuth et al., 2007; Weingart, 2007; Schnedermann et al., 2018).

At room temperature, the ca 35 kcal of excitation energy stored in bathorhodopsin (Cooper, 1979) drives further relaxation via several intermediates until the metarhodopsin IIa-IIb equilibrium is reached within ms. This relaxation process subtly rearranges chromophore, protein residues and H-bonded networks up to the metarhodopsin stage, where the Schiff base transfers its proton, the counterion and another Glu at the intracellular side of the protein become protonated and an interhelical activity switch reshuffles helical segments to open up binding residues for the G-protein (Hofmann, 2000; Vogel et al., 2007; Vogel et al., 2008; Pope et al., 2020). The chromophore is subsequently slowly released via hydrolysis of the Schiff base to generate the nearly inactive apoprotein opsin (Wald, 1953; Rothschild et al., 1987; Jastrzebska et al., 2011). *In vivo* the active state is rapidly inactivated through phosphorylation and arrestin binding, however, which blocks activation of the G protein (Ranganathan and Stevens, 1995).

The photochemical profile of other monostable pigments (human rod rhodopsin, several cone pigments) has been investigated to much less depth, but is quite comparable to the bovine rod pigment (Barry and Mathies, 1987; Kusnetzow et al., 2001; Hofmann and Palczewski, 2015; Kazmin et al., 2015). However, the kinetics differ somewhat. For instance, the investigated cone pigments show more rapid kinetics in most steps (Imai et al., 1997; Vissers et al., 1998; Chen et al., 2012; Sato et al., 2012). Ultra-violet absorbing cone pigments may be more complex, as photoisomerization is accompanied by protonation of the Schiff base (Kusnetzow et al., 2004; Mooney et al., 2012).

The photochemical profile of bistable pigments, investigated thus far (squid, octopus and some insect pigments), follow a scheme similar to the monostable pigments up through formation of the M-intermediate and with comparable kinetics (Figure 8B) (Gärtner, 2000; Stavenga et al., 2000; Vought et al., 2000; Murakami and Kouyama, 2015). It is reported that in cephalopods the M-intermediate in fact forms a pH-dependent equilibrium between a protonated (acid M) and a deprotonated state (alkaline M). This involves the Schiff base of the chromophore, and the alkaline M is strongly blue-shifted (Liang et al., 1994; Vought et al., 2000). Photo-reisomerization of the M state to the original “dark” state is again quite efficient with a quantum yield around 0.4 (Stavenga et al., 2000).

### Type-1 Family

The “dark” state of type-1 pigments contains a chromophore with the all-*trans,* 15-*syn* configuration (Figure 1). Rapid spectroscopy has been performed on quite a number of type-1 pigments, and the global scheme is quite similar to that of bacteriorhodopsin (Figure 8C). However, the kinetics of the slower steps (M and subsequent ones) and thereby the overall cycle time can vary considerably from ms up to minutes (Rozenberg et al., 2021; Tsunoda et al., 2021; Broser, 2022; Nagata and Inoue, 2022).

Out of all rhodopsins the photochemistry of BR is understood in most detail (Wickstrand et al., 2015; Nango et al., 2016). Femtosecond XFEL crystallography has even revealed very early responses to photoexcitation of the chromophore (Nogly et al., 2018). The adjacent protein residues and water molecules already react to the charge delocalization in the excited chromophore before the isomerization is initiated (Tahara et al., 2019a). During the isomerization process more of the protein environment becomes involved while the chromophore rapidly relaxes along a 2-state trajectory on the excited state potential surface to a conical intersection, where it effectively crosses in ca 500 fs over to a ground state energy surface into a “hot” transient hybrid state (J) and then relaxes thermally in about 3 ps into the photoproduct K, which contains a still significantly twisted 13-*cis*, 15-*anti* chromophore, but is stable below 150 K (Lanyi, 2004). Here, a major driving force is the elongation of the C13-C14 bond in the excited state in combination with electrostatic re-arrangement and weakening of the hydrated H-bonded network in the Schiff base region. At room temperature, the ca 15 kcal of excitation energy stored in K (this can be higher in sensory rhodopsins) (Birge et al., 1991; Govorunova et al., 2017; Rozenberg et al., 2021) drives further relaxation via the spectrally distinguishable L intermediate until the M states are reached in ca 50 µs. This relaxation process again subtly re-arranges chromophore, protein helices and H-bonded networks up to the M states, where the Schiff base transfers its proton via a water molecule to the counterion and the hydrated H-bonded network opens up a proton gateway to the extracellular membrane surface. The M-states thermally decay via several intermediates in tens of ms to the BR ground state, during which the Schiff base is reprotonated via proton transfer from residue Asp96, a proton is taken up from the intracellular surface and the chromophore is re-isomerized to the all-*trans*, 15-*syn* configuration. In fact, all-*trans* is the most stable configuration for free retinal (Ganapathy and Liu, 1992). Nevertheless, in some archaeal rhodopsins including BR the chromophore slowly enters an all-*trans*, 15-*anti ↔ 13*-*cis*, 15-*syn* equilibrium when stored in the dark (dark adaptation). The latter chromophore is photo-excited in the light and via a separate non-productive photocycle rerouted to the ground state BR (Smith et al., 1989; Oesterhelt et al., 1991). In channelrhodopsins the opposite phenomenon is observed, where prolonged illumination reduces the activity, since an equilibrium between pigments with an all-*trans,* 15-*anti* and a 13-*cis*, 15-*syn* chromophore configuration is generated (light-adaptation with partial desensitization) (Bruun et al., 2015; Kuhne et al., 2019; Rozenberg et al., 2021; Govorunova et al., 2022b).

Using serial synchrotron crystallography, the slower conformational changes from 5 to ca 40 ms were recorded in the BR photocycle and involve small α-helical rearrangements, chromophore re-isomerization and proton uptake, ending in formation of the ground state (Weinert et al., 2019). A very recent study using advanced high-resolution atomic force spectroscopy at the single-molecule level investigated the BR photocycle after M formation (Perrino et al., 2021). It was concluded that a cytoplasmic gate for proton uptake opens up at about 3 ms after photo-excitation lasting for about 14 ms. Surprisingly, this same study observes a “black-out period” of tens of ms before a recycled ground state can be photo-reactivated. This uncovers a very interesting new phenomenon reminiscent of comparable nonresponsive states in animal voltage-regulated channels (Armstrong, 1992). Meanwhile, XFEL studies have also been performed on other ion pumps and channels. A femtosecond XFEL study of the sodium-pumping rhodopsin from *Krokinobacter eikastus* (KR2) again observed photo-isomerization of the chromophore to start in the femtosecond range and completed within 2 ps (Skopintsev et al., 2020). Changes in the local structure of the binding site and early conformational changes in the protein backbone are observed in the early nanosecond range. Further subtle rearrangements result in Schiff base deprotonation in µs and in the early ms range a gate opens up and transient binding of a Na ^+^ ion in the vicinity of the Schiff base is observed with release within 20 ms. A femtosecond XFEL study of the chloride pump from the flavobacterium *Nonlabens marinus* follows the conformational adaptations between 1 and 100 ps after photo-excitation (Yun et al., 2021). It shows the final rearrangements of the chromophore to the 13*-cis* configuration within 50 ps, together with the dynamics of the hydrated H-bonded network and deformations in the local α-helical elements. Following chromophore isomerization the chloride ion first dissociates from the protonated Schiff base and then starts to diffuse away. Additional molecular details of the interactions and trajectory of the chloride ion are provided by recent ps up to ms studies using time-resolved serial crystallography in combination with spectral and theoretical analysis (Hosaka et al., 2022; Mous et al., 2022). An XFEL study of the channelrhodopsin chimera C1C2, that photochemically behaves like ChR1, investigated the photo-induced conformational changes from 1 µs to 4 ms (Oda et al., 2021). Photo-isomerization induces a kink in the chromophore structure, triggering shifts in the retinal binding lysine residue and TM7, starting at around 1 µs and increasing during formation of the M-state up to 4 ms. This induces small lateral shifts of the chromophore and in TM7 and TM3 at around 50 µs. It is postulated that these rearrangements forebode the subsequent opening of the gates in the cation channel pore, although these were not observed in the crystal. The XFEL and serial crystallography studies beautifully illustrate the powerful but subtle design and the broad potential of the photo-driven nanomachinery. Less detailed studies basically show a similar pattern (Table 4). Subtle differences in early kinetics and conformational adaptation in chromophore and adjacent protein elements following photo-excitation are observed in the ultrarapid studies. A cautious interpretation could be that the structure of the hydrated H-bonding network in the complex counterion is an important roadmap for the light-triggered protein activity, which also depends on the pKa of the direct counterion (Hontani et al., 2017b; Oda et al., 2021; Chang et al., 2022).

In this context it should be realized that crystal structures have their limitations (García-Nafría and Tate, 2020; Guo, 2020). Detergent exposure may affect elements of the protein structure, and the crystal will certainly constrain larger conformational alterations in the protein, which may occur in the slower phase of the photocycle (Weinert et al., 2019; Oda et al., 2021; Govorunova et al., 2022b). Hence, it would be preferable to study the slower photocycle phases with experimental approaches that can handle membrane-bound systems as shown in Figure 4, like time-resolved AFM, cryo-EM and vibrational spectroscopy.

The general scheme for the photocycle of BR (Figure 8C) also holds for other type-1 pigments, though the kinetics after M formation can vary significantly (Wand et al., 2013; Tahara et al., 2015; Han et al., 2020; Smitienko et al., 2021). The decay is much slower for sensory rhodopsins, enzyme-rhodopsins and heliorhodopsins, possibly since longer interaction with their cognate partner is required for regulated signal transduction. In fact, some sensory rhodopsins and enzyme-rhodopsins exhibit a bistable photocycle (Kawanabe et al., 2007; Broser et al., 2020) and proton transfer to the counterion may not occur (Bergo et al., 2006).

## Bioengineering

This section samples the impressive expansion in the field of rhodopsins bioengineered by creative exploitation of their design principles. Often, similar strategies are utilized for both type-1 and type-2 pigments, and therefore they are clustered together in the following subsections.

### Shifts in Spectral And/or Functional Properties

#### Chromophore

Very early on in the 1960s, it was realized that the beautiful design and versatility of rhodopsins could be studied and exploited by modifying the chromophore and changing the spectral properties (Blatz et al., 1969; Kropf et al., 1973). Since protein modeling was not really established at that time, this led to a surge of trial-and-error synthetic efforts to test a large number of retinal analogs on their ability to incorporate into the binding site and to modulate spectral and/or functional properties (Balogh-Nair and Nakanishi, 1982; Derguini and Nakanishi, 1986; Liu and Asato, 1990; Crouch et al., 2002). Initially, this was mainly performed on bovine rod rhodopsin and bacteriorhodopsin, which were easily isolated in sufficient quantities. In this way both bathochromic and hypsochromic spectral shifts up to ca 80 nm could be realized, frequently with retardation of photo-kinetics or total loss of function. For instance, using “locked” retinals (blocking functional photo-transformations) it was confirmed that the photo-isomerization process was essential for the functionality and that the ring-polyene chain connection was 6-s-*cis* in type-2 rhodopsins and 6-s-*trans* in type-1 pigments (Figure 1) (Crouch et al., 1984; Fukada et al., 1984; Harbinson et al., 1985; van der Steen et al., 1986; DeGrip et al., 1990; Bhattacharya et al., 1992a; Ganapathy et al., 2015). Also, the remarkable observation was made with bovine opsin, that next to the 11-*cis* and 9-*cis* retinal, also the 7-*cis*, 7, 9-di*cis*, and 7, 9, 13-tri*cis* retinal isomers could form a functional pigment, inducing a 40–50 nm blue-shift but reducing thermostability (DeGrip et al., 1976; Liu et al., 1984). In general, it turned out that the bovine opsin binding pocket could better accommodate more voluminous modifications than the bacterio-opsin pocket, suggesting a more constrained character for the latter one. This was later validated in 3-D structures, but other type-1 pigments or photo-intermediates can be less selective (Popp et al., 1993; Inoue et al., 2012; Mori et al., 2013). New analogs are still frequently generated, in particular because recombinant production of mutated opsins modifies the binding pocket constraints. In addition, protein modeling has become more straightforward and for optogenetics larger spectral shifts and other functionalities like higher photosensitivity or higher fluorescence yields are in demand (see below).

#### Protein Joins In

Once recombinant DNA technology allowed the production of functional opsins in heterologous hosts, one could use this technology to adapt the intrinsic potential of opsins to one’s need and design. Combining synthetic retinal design with recombinant DNA opsin modification opened up a marvelous toolbox to investigate the structure and functional mechanism of rhodopsins as well as to probe new functionalities and applications. This trend is evolving more and more rapidly. Initially, binding site residues were modified to probe their contribution to the packing, stabilization and spectral tuning of the chromophore (Khorana et al., 1987; Sakmar et al., 1989; Nathans, 1992; Sakmar and Fahmy, 1995; Giesbers et al., 2007). A salient example is the accumulating evidence for type-1 pigments, that three positions around the retinal chromophore, corresponding to L93, P186 and Ala215 in BR, function as natural spectral-tuning modules that systematically shift the absorbance spectrum of the chromophore without affecting molecular function (Table 5). This further inspired detailed analysis with modified and/or labeled retinals (^13^C, ^2^H, F) and protein residues (^13^C, ^15^N, F, azido, spin labels) using fluorescence, vibrational, EPR and NMR spectroscopy (Table 5). This profited from as well as steered development of sophisticated theoretical and *in-silico* procedures, like DFT, QM/MM and molecular dynamics (Altun et al., 2008; Collette et al., 2018; Del Carmen Marín et al., 2019b; Dokukina et al., 2019; Pieri et al., 2019; Shao et al., 2020; Nikolaev et al., 2021; Scholz and Neugebauer, 2021; Shen et al., 2021; Pedraza-González et al., 2022). All these elements have already profoundly deepened our insight into the structure and mechanism of bovine rod rhodopsin and bacteriorhodopsin, the frontrunners of type-2 and -1, respectively. However, more members are following up. Some recent conspicuous examples are mentioned in the next section.

**TABLE 5:** Selected additional citations for the section “Bioengineering”.

Subsection
Chromophore
*Type-1 pigments*: Balogh-Nair and Nakanishi, (1982); Muradin-Szweykowska et al. (1984); López et al. (2005); Sineshchekov et al. (2012); AzimiHashemi et al. (2014); Ganapathy et al. (2015); Mei et al. (2018); Ganapathy et al. (2019); Hontani et al. (2019); Munro et al. (2019); Chuon et al. (2021)
*Type-2 pigments:* Arnaboldi et al. (1979); Mollevanger et al. (1987); Friedman et al. (1989); Bhattacharya et al. (1992b); Feng et al. (1997); Huang et al. (1997); DeLange et al. (1998a); Iwasa et al. (1998); Lugtenburg et al. (1999); Verdegem et al. (1999); Wada et al. (2000); Spooner et al. (2004); Wang et al. (2004); Hirano et al. (2006); Verhoeven et al. (2006); DeGrip et al. (2007); Concistrè et al. (2008); Aguilà et al. (2009); Bovee-Geurts et al. (2009); DeGrip et al. (2011); Srinivasan et al. (2014); Alexander et al. (2017); Bovee-Geurts et al. (2017); Buda et al. (2017)
Protein
*Type-1 pigments*
Spectral properties: Alexiev et al. (2000); Béjà et al. (2001); Hayashi et al. (2001); Shimono et al. (2001); Bielawski et al. (2004); Mori et al. (2013); Ozaki et al. (2014); Ganapathy et al. (2015); Agathangelou et al. (2018); Oda et al. (2018); Singh et al. (2018); Ganapathy et al. (2019); Inoue et al. (2019); Kuhne et al. (2019); Kojima et al. (2020d); Nakajima et al. (2021); Tsujimura et al. (2021); Shim et al. (2022)
Vibrational spectroscopy: Sonar et al. (1995); Amsden et al. (2007); Ikeda et al. (2007); Yi et al. (2016); Tomida et al. (2020)
NMR/EPR spectroscopy: Steinhoff et al. (1995); Griffiths et al. (2000); Herzfeld and Lansing, (2002); Maly et al. (2008); Shi et al. (2009); Wang et al. (2013); Becker-Baldus et al. (2015); Brown and Ladizhansky, (2015); Shigeta et al. (2017); Azadi-Chegeni et al. (2018); Kaur et al. (2019); Lavington and Watts, (2020); Kawamura et al. (2021); Tomida et al. (2021)
Crystallography/EM: Volkov et al. (2017)
Other: Khorana et al. (1987); Steward and Chamberlin, (1998)
*Type-2 pigments*
Spectral properties: Nakayama and Khorana, (1991); Chan et al. (1992); Asenjo et al. (1994); Yokoyama, (1995); Hope et al. (1997); Dunham and Farrens, (1999); Kochendoerfer et al. (1999); Hunt et al. (2001); Alexiev et al. (2003); Piechnick et al. (2012); Devine et al. (2013); McKee et al. (2021)
Vibrational spectroscopy: Haris et al. (1992); Lin et al. (1992); DeLange et al. (1998b); Lin et al. (1998); Ye et al. (2009); Rothschild, (2016)
NMR/EPR spectroscopy: Smith et al. (1996); Creemers et al. (1999); Creemers et al. (2002); Eilers et al. (2002); Hubbell et al. (2003); Werner et al. (2007); Altenbach et al. (2008); Hornak et al. (2010)
Computational: Nielsen, (2009); Collette et al. (2018); Peters et al. (2020)
Other: Yokoyama, (2000)
Conversion: Berndt et al. (2014); Vogt et al. (2015); Inoue et al. (2016)
Optogenetics
*Type-1 pigments*: Tsunoda et al. (2006); Airan et al. (2009); Erbguth et al. (2012); Sudo et al. (2013); Wietek et al. (2015); Alfonsa et al. (2016); Berglund et al. (2016); Berndt et al. (2016); Kulkarni and Miller, (2017); Brown et al. (2018); Pediani et al. (2018); Piatkevitch et al. (2018); Xu et al. (2018); Alabugin, (2019); del Carmen MarÍn et al. (2019a); Marshel et al. (2019); Jun and Cardin, (2020); Milosevic et al. (2020); Baillie et al. (2021); Hayashi et al. (2021); Kathe et al. (2021); Nakao et al. (2021); Panzer et al. (2021); Zhou et al. (2021); Govorunova et al. (2022a); Guo et al. (2022); Li et al. (2022); Nakao et al. (2022); Shim et al. (2022); Yaguchi et al. (2022)
*Type-2 pigments:* De Silva et al. (2017); Patriarchi et al. (2018); Berry et al. (2019); Owen et al. (2019); Copits et al. (2021); Hickey et al. (2021); Banskota et al. (2022)
Cell factories: Charvolin et al. (2009); Kim et al. (2012); Schlinkmann and Plückthun, (2013); Pinhassi et al. (2016); Lips et al. (2018); Pérez et al. (2019a); Konno et al. (2021); Polito et al. (2021); Zhang et al. (2021); Fujiyabu et al. (2022)

#### Conversion

The manipulations described in the previous subsection frequently revealed surprising conversions in activity profile, exemplifying the versatile design principle of the rhodopsins (Kaneko et al., 2017). An interesting example is presented by the *Nonlabens marinus* inward chloride pump NMR-3 and the *Krokinobacter eikastus* sodium exporter KR2 (Hosaka et al., 2016; Yun et al., 2020). With only 35% sequence identity, the crystal structures are remarkably similar, but the gating residues for Cl^−^ and Na^+^ are located at the opposite site of the membrane (Kato et al., 2015a; Hosaka et al., 2016; Kovalev et al., 2019; Yun et al., 2020). Another example is the huge mutagenesis effort that converted a thermophilic rhodopsin into the best thermally stable rhodopsin available to date, while retaining pump activity (Yasuda et al., 2022). On the other hand, selective mutations in the opsin could convert BR into an inward chloride pump, the sodium pump KR2 into a selective light-driven cation channel, the proton pumps Archaerhodopsin-3 (AR3) and *Coccomyxa subellipsoidea* rhodopsin (CsR) into light-driven proton channels, and the proton pump GR from *Gloeobacter violaceus* into a fluorescent chloride sensor (Sasaki et al., 1995; Brown et al., 1996; Inoue et al., 2015; 2016; Fudim et al., 2019; Vogt et al., 2019; Tutol et al., 2021). Alternatively, a cyanobacterial chloride pump could be converted into a proton pump (Hasemi et al., 2016; Kikukawa, 2021). Novel retinal A1 and A2 analogs with an elongated polyene chain (10 instead of 9 carbons) still could incorporate into the binding pocket of the ReaChR channelrhodopsin inducing red-shifts up to ca 30 nm (Okitsu et al., 2020). However, when tested upon AR3, one A2 analog induced a 41 nm blue-shift and again converted it into a light-driven proton channel (Takayama et al., 2018). Another novel retinal analog (MMAR, Figure 5.) smoothly incorporated into the binding pocket of the proton pump Green Proteorhodopsin (GPR), inducing a 47 nm red-shift, but when combined with a Phe →Ser mutation near the binding pocket, an unprecedented 200 nm red-shift was observed (Ganapathy et al., 2017). This retinal analog not only maintains some pump activity under near-infrared illumination (700–900 nm region; NIR), but also induces strong fluorescence emission in the NIR, probably emitted in the first picoseconds after excitation (Hontani et al., 2018; Mei et al., 2018; 2020). Proton-pumping rhodopsins in several eubacteria (XR, GR and TR) harbor a carotenoid derivative (salinixanthin) close enough to act as an antenna and transfer electronic excitation to the retinal (Balashov et al., 2010; Imasheva et al., 2011; Misra et al., 2019; Jana et al., 2020). This combination significantly broadens the spectral sensitivity of the rhodopsins for blue wavelengths, and the carotenoid binding option can also be introduced into other pigments (Anashkin et al., 2018). Attempts have also been made to generate chimeric pigments with combined functionality. The earliest example was a BR mutant containing loops of rod rhodopsin being able to weakly activate the G protein (Geiser et al., 2006). This concept in BR was further developed (Sasaki et al., 2014; Kaneko et al., 2017; Yoshida et al., 2017) and also found wider application in other rhodopsins (Kaneko et al., 2017). Chimeras could be produced between type-1 and type-2 pigments, often with shared properties and variable potential for G protein activation (Kojima et al., 1996; Geiser et al., 2006; Airan et al., 2009; Nakatsuma et al., 2011; Sasaki et al., 2014; Bedbrook et al., 2017a; Kaneko et al., 2017; Hickey et al., 2021). A remarkable example is that the C1C2 chimera could be crystallized and a high-resolution crystal structure obtained long before its “parent” channelrhodopsins ChR1 and ChR2, (Kato et al., 2012). In a sequel, new chimeric channelrhodopsins with better performance were generated using structure-guided recombination (Bedbrook et al., 2017a). The chimeric concept has also resulted in type-2 recombinants with variable success (Kojima et al., 1996; Giesbers et al., 2007; Hickey et al., 2021).

These selected examples, along with some more references collected in Table 5, already give an impression of the fabulous potential and prospects of the rhodopsin clan. The most impressive flux, however, is noticeable in the optogenetics field.

#### Optogenetics

Neuronal activity and circuitry are of the essence for multicellular life. Much effort is dedicated to studying activity regulation and circuitry in complex tissues like the brain. This used to be a highly challenging electrophysiological operation, requiring invasive electrodes and precise surgical location. Once it was realized, that rhodopsins could be properly expressed in animal tissues with genetic targeting to specific neurons using selective promoters, it became possible to monitor and regulate neuronal activity by light using endogenously expressed rhodopsins (Boyden et al., 2005). This led to an explosion of research activity in a new field, coined optogenetics (Deisseroth, 2010, 2015; Rost et al., 2017; Kandori, 2020; Friedman, 2021). Initially, only type-1 rhodopsins were considered, since ion fluxes can directly modulate neuronal activity. Also, all-*trans* retinal is intrinsically available in animal cells and type-1 pigments complete a full photocycle.

In a first breakthrough, a cation-selective channelrhodopsin originally identified in *Chlamydomonas reinhardtii* termed ChR2 (Nagel et al., 2003) was exploited. ChR2 was shown to elicit action potentials in cultured neurons upon illumination (Boyden et al., 2005; Cardin et al., 2010; Klapoetke et al., 2014; Berndt et al., 2016; Deisseroth and Hegemann, 2017). This domain rapidly expanded into ion pumps, which can activate or silence neuronal activity (Chow et al., 2010). Simultaneously, pigments were modified to change spectral range, increase current output, alter photo- and response kinetics, improve membrane targeting, etc. (Lin et al., 2013; Kushibiki et al., 2014; Kato et al., 2015b; Brinks et al., 2016; Govorunova et al., 2017; Cho et al., 2019; Krol et al., 2019; Kojima et al., 2020b; Gong et al., 2020). Eventually, enzyme-rhodopsins as well as bistable type-2 pigments also entered the field, being able to modulate cellular metabolic processes up to gene expression (Mukherjee et al., 2019; Karapinar et al., 2021; Mahn et al., 2021; Rodgers et al., 2021; Tsunoda et al., 2021; Vierock et al., 2021). Bistable type-1 and -2 pigments allow further control, since their activity is triggered by illumination, but ends near the M(eta) stage, which can be photoreversed by illumination in another spectral range (Sheves and Friedman, 1986; Koyanagi and Terakita, 2014; Mederos et al., 2019; Eickelbeck et al., 2020).

A second breakthrough came with the discovery that the intensity of the fluorescence emission of the proton pumps GPR and AR3, be it quite weak, is modulated by the membrane potential (Kralj et al., 2011; Saint Clair et al., 2012b; Kralj et al., 2012). This triggered another burst of research dedicated to improve the voltage sensing of these pumps (minimizing pump activity, shifting spectral range, improving quantum yield, voltage sensing potential, temporal resolution, etc.) by a range of technologies like directed and scanning mutagenesis, multidimensional directed evolution, library screening and machine learning (McIsaac et al., 2014; Engqvist et al., 2015; McIsaac et al., 2015; Abdelfattah et al., 2016; Karasuyama et al., 2018; Kojima et al., 2020a). This was initially mostly performed on AR3, generating a whole family of mutants with different response characteristics (Quasar1 to 3, pa-Quasar3, Novarch, Archon1 and 2, Arch-EEN, Quasar6, Somarchon to name a few) (Piatkevich et al., 2019; Chien et al., 2021). The fluorescence of these voltage sensors most likely originates in late-stage photo-intermediates (Maclaurin et al., 2013). The introduced mutations may even result in a complex bistable photo-equilibrium between a fluorescent and a non-fluorescent state (Mei et al., 2021; Penzkofer et al., 2021). Meanwhile a host of additional voltage sensors have been developed. Next to optimized rhodopsins and chimeric rhodopsin fusions, fusion proteins of light-sensitive opsin cores with other fluorophores, often GFP derivatives or synthetic dyes, and of other voltage sensors with fluorescent rhodopsins have become popular (Bando et al., 2019; Kannan et al., 2019; Lee et al., 2019; Berglund et al., 2020; Zhang X. M. et al., 2021).

Further control has been sought by combining optogenetics with classical electrophysiology (electro-optogenetics) or combining voltage sensors and neuronal activators and/or silencers both based on rhodopsins (all-optical electrophysiology) (Hochbaum et al., 2014; Afshar Saber et al., 2018; Sridharan et al., 2022). In the latter case, it is important to separate the spectral sensitivities to allow selective control and avoid optical cross-talk. In addition, much effort has been put into shifting the spectral range of the optogenetic tools and sensors as far as possible into the NIR, since NIR radiation penetrates much further into the mammalian brain (up to cm compared to several mm for e.g. blue-green light) (Larkum et al., 2018; Govorunova et al., 2020; Broser, 2022). For this purpose, mutagenesis of far-red absorbing rhodopsins like Crimson and CrimsonSA would be a good starting point (Oda et al., 2018). Another option is the novel channelrhodopsin ChRmine, which has quite unusual properties, including a trimeric structure similar to BR (Marshel et al., 2019; Kishi et al., 2022). A very fascinating example is NeoR, a subunit in the heterodimeric rhodopsin-cyclase from the fungus *Rhizoclosmatium globosum.* NeoR is quite exceptional, as it harbors three carboxyl residues near the chromophore and has an absorbance maximum at 690 nm with strong fluorescence emission at 707 nm (Broser, 2022). Other gateways could include special optical technologies or local NIR-converting nanoparticles and two-photon spectroscopy, which are more complicated (Sneskov et al., 2013; Chen, 2019; Matarèse et al., 2019; Yu et al., 2019; Adesnik and Abdeladim, 2021; Lehtinen et al., 2022), or designing special retinal analogs. The latter was quite successful, shifting absorbance maxima up to ca 750 nm with fluorescent emission around 800 nm using merocyanine analogs or MMAR (Figure 5) (Derguini et al., 1983; Hoischen et al., 1997; Liu and Asato, 2003; Herwig et al., 2017; Hontani et al., 2018; Mei et al., 2020). The strong red-shift in these analog chromophores, as well as in the A1 chromophore in NeoR is contributed to extensive delocalization of the positive charge from the protonated Schiff base over the polyene element (Figures 1, 5) (Liu and Asato, 2003; Lutnaes et al., 2004; Ganapathy et al., 2019; Broser, 2022). This will strongly reduce the energy gap between the ground and first excited state. Incorporation of retinal A2 into NeoR-opsin already effectuates a further 69 nm red-shift (Broser et al., 2020). Hence, it would be very interesting to investigate whether the combination of NeoR-opsin or mutants with bathochromic analogs like MMAR would even further red-shift the absorbance band and increase the gap with the emission band. Optogenetic application, however, requires invasive administration of the retinal analog and may need transient depletion of the endogenous A1.

So far, the field of optogenetics has progressed spectacularly, from neuron and brain slice cultures, up to intact animals including insects, *C. elegans,* mice and macaques (Bi et al., 2006; Flytzanis et al., 2014; Inagaki et al., 2014; AzimiHashemi et al., 2019; Babl et al., 2019; Piatkevich et al., 2019; Gong et al., 2020; Wagner et al., 2021; Wright et al., 2021) and is being extended to human disease models (Wright et al., 2017; Williams et al., 2019; Córdova et al., 2021; Fougère et al., 2021; Lindner et al., 2021). Future prospects will be touched upon in the next section.

#### Cell Factories

While rhodopsins drive important physiological processes in prokaryotes and eukaryotes, and can contribute significantly to the energy requirement of their hosts, implementing this into biotechnological resources like cell factories has not yet developed very far (Walter et al., 2010). *E. coli* can profit from expression of a rhodopsin proton pump (Martinez et al., 2007; Choi et al., 2014; Na et al., 2015; Wang et al., 2015; Kim et al., 2017; Song et al., 2020). However, the extent to which this can for instance support production of useful consumables or commodity chemicals needs to be established. Cyanobacteria like *Synechocystis* sp. PCC6803 and *Synechococcus* already exploit chlorophyll-based oxidative photosynthesis to gather solar energy and are under intense investigation as cellular factories (Wijffels et al., 2013; Angermayr et al., 2015; Du et al., 2018; Knoot et al., 2018; Carpine et al., 2020). They do not have an endogenous opsin, but do produce all-*trans* retinal and can serve as a heterologous host for expression of rhodopsin proton pumps (Chen et al., 2016b; Chen et al., 2017; Chen et al., 2019a). Expression of these pumps was considered as a potential extra energy source, but the contribution of these pumps towards cellular energy production appeared to be limited (Chen et al., 2019b). This may be due to the metabolic constraint of proton fluxes, and/or to the chlorophylls and carotenoids absorbing much of the incoming radiation up to ca 650 nm (the PAR region). Attempts to express the GPR F234S mutant in combination with the retinal analog MMAR were successful in generating a proton pump absorbing in the 700–800 nm range, outside the PAR region. However, this still did not generate sufficient additional energy due to the lower pump activity of this mutant and failed to sustain bacterial growth under NIR illumination (Chen et al., 2018).

## Prospects

A major asset of the rhodopsin family is the impressive versatility of the design principle: a relatively simple photosensitive ligand, constrained to allow selective photoisomerization with a high quantum yield, triggering subtle but effective conformational changes in the protein opening up specific binding sites or ion transport pathways.

*Genome mining* will undoubtedly discover new type-1 and type-2 or related variants, especially considering the still vast reservoir of unexplored microbial and invertebrate life forms. For instance, the apparent non-photic activity of (rhod)opsins in certain physiological conditions (thermo-, mechano- or chemo-sensing) may add a new chapter to this family saga (Leung and Montell, 2017; Katana et al., 2019; Baden et al., 2020; Fleming et al., 2020; Hasegawa et al., 2020; Mei et al., 2020; Zabelskii et al., 2021; Feuda et al., 2022). Next to that, insight into the effect of pathological mutations will become an ever more important asset in medical diagnostics and potential treatment. This has already been widely explored in the case of rod rhodopsin and retina-degenerative diseases, (Athanasiou et al., 2018). Expression and functional and structural characterization of new (rhod)opsins or mutants still involves an elaborate effort, but this may be considerably mitigated soon.

The phenomenal progress in *artificial intelligence and machine learning* already culminated in the design of software packages like RoseTTAFold and Alphafold, that are quite successful in predicting the protein fold from the primary sequence (Humphreys et al., 2021; Jumper et al., 2021). Considering the respectable number of crystal structures for type-1 pigments and G protein-coupled receptors already obtained, this *in silico* approach will be of invaluable help to close in on the 3-D structures of rhodopsin sequences identified to date, as well as those yet to be identified. A similar track is conceivable for the assessment of spectral and functional properties. Experimental analyses, in combination with *in-silico* techniques like DFT, machine learning and quantum-chemical computing already made big strides in establishing the contribution of individual opsin residues and water molecules to the spectral tuning of rhodopsins (Kato et al., 2015; Bedbrook et al., 2017b; Karasuyama et al., 2018; Bedbrook et al., 2019; Nikolaev et al., 2020; Inoue et al., 2021; Yang et al., 2022). However, this approach always requires 3-D information. It would be very desirable to build in additional functionalities, e.g. to predict an approximate absorbance maximum, into the sequence-to-structure software packages. This could then be easily expanded towards predicting the effect of mutations and the fit and effects of retinal derivatives or even more distant chromophores. Suggestions for functionality (specific pump or channel, enzymatic domains, thermal stability) probably could also be in reach, though mechanistic details (photoisomerization process, quantum yield of isomerization or fluorescence emission, early conformational changes) may be aiming too high.

Such developments will be a goldmine for *optogenetics*. Rapid prediction of spectral and functional properties and optimal targeting of desired mutants would be very valuable. Likewise, assessment of new constructs like chimeric pigments, fused monomers, oligomeric assemblies, enzyme activating pigments, new signaling partners and the like can be set up *in silico* and will require much less experimental justification (Sasaki et al., 2014; Abdelfattah et al., 2020). This would undoubtedly be accompanied by further physiological expansion of optogenetic tools. A wider spectral range of neuronal activity modulators and voltage sensors together with improved optics will increase the scope for (all)-optical electrophysiological characterization of neural circuitry, also lending insight into neuronal function (and dysfunction) in the brain (Villette et al., 2019; Guimarães Backhaus et al., 2021; Sharma et al., 2021; Zou et al., 2021; Prakash et al., 2022; Sridharan et al., 2022; Tan et al., 2022). Other important medical targets may also arise using optogenetics to correct physiological defects and address pathological conditions, where first steps have already been taken (Braun et al., 1995; Deubner et al., 2019; Shen et al., 2020; Acharya et al., 2021; Cokic et al., 2021; Kathe et al., 2021; Gilhooley et al., 2022; Sun et al., 2022).

Several concepts to utilize rhodopsins in *bioelectronic and biomimic nanotechnology* have already been attempted, but did not yet really come to maturation (Khodonov et al., 2000; Kuang et al., 2014; Hirschi et al., 2019; Aprahamian, 2020; Shim et al., 2021). With the rapidly growing insight in the structural and mechanistic potential of the rhodopsin pigments, this is expected to change at short notice. So far, electro-optical phenomena have been investigated in 2D crystals, lipid films and other matrices (Oesterhelt et al., 1991; Miyasaka et al., 1992; Hong, 1994; Wagner et al., 2013; Zhao et al., 2015; Ji et al., 2017; Gruber et al., 2022). With help of the above mentioned software packages, the design of specific constructs with high performance and stability under the system’s conditions will be facilitated.

This would also be the case for application in *cell factories*. The most interesting and rewarding application in this respect is the notion of “synergistic photosynthesis,” the combination of chlorophyll-based oxidative photosynthesis with retinal-based phototrophy, using high-performance rhodopsin proton pumps absorbing in the NIR (Chen et al., 2016a; Chen et al., 2019b). This will also require adaptation of proton regulation in the host cell or introduction of special cellular organelles containing the pump and an ATP-synthase. In eukaryotic cells like algae or fungi, targeting of a proton pump to mitochondria to increase ATP levels for production of commodity chemicals under selected conditions can be further developed (Hoffmann et al., 1994; Hara et al., 2013; Tkatch et al., 2017; Imai et al., 2019; Berry and Wojtovich, 2020). In general, designing highly active ion pumps absorbing in the 700–800 nm region, i.e. outside the PAR region, is essential for productive “synergistic photosynthesis.” Again, artificial intelligence can be a decisive factor here.

## Epilogue

In roughly 10 years, the rhodopsin field has reached a century’s worth of experimental investigation. In this review, we have mainly touched upon the surface of the phenomenal development in this field, somewhat like molecular force microscopy. In the coming 10 years we expect its expansion to continue and to eventually require an at least ten-volume book series for full documentation. By that time, we will hopefully have a better understanding of how a selection of twenty amino acids can lead a membrane protein domain of 300–400 amino acids surrounding a small chromophoric group to such mechanistic versatility.

## Abbreviations

AFM, Atomic force microscopy; AR3, Archaerhodopsin-3; BR, Bacteriorhodopsin; C1C2, chimera between channelrhodopsin-1 and -2; Cryo-EM, Cryo-electron microscopy; CTAB, Cetyltrimethylammonium bromide; DDM, Dodecylmaltoside; DFT, Density functional theory; DPC, Dodecylphosphocholine; EPR, Electroparamagnetic resonance; FTIR, Fourier-transform infra-red; GR, *Gloeobacter violaceus* rhodopsin; LDAO, Lauryldimethylaminoxide; MSP, Membrane scaffold protein; NG, Nonylglucoside; OG, octylglucoside; PAR, Photosynthetically active region; PM, plasma membrane; RGR, RPE-retinal G protein-coupled receptor; RPE, Retinal Pigment Epithelium; SMA, Styrene-maleic-acid-copolymer; TR, Thermophilic rhodopsin; TR-WAXS, Time-resolved wide-angle X-ray scattering; XFEL, X-ray free electron laser.
